# Lactic Acid Fermentation to Re-cycle Apple By-Products for Wheat Bread Fortification

**DOI:** 10.3389/fmicb.2019.02574

**Published:** 2019-11-06

**Authors:** Vincenzo Cantatore, Pasquale Filannino, Giuseppe Gambacorta, Ilaria De Pasquale, Stefan Pan, Marco Gobbetti, Raffaella Di Cagno

**Affiliations:** ^1^Department of Soil, Plant and Food Science, University of Bari Aldo Moro, Bari, Italy; ^2^PAN Surgelati Srl, Bolzano, Italy; ^3^Faculty of Sciences and Technology, Libera Università di Bolzano, Bolzano, Italy

**Keywords:** apple by-products, fermentation, hydration properties, dietary fibers, wheat bread fortification

## Abstract

Apple by-products (ABP) underwent fermentation (48 h at 30°C, Fermented-ABP) with a selected binary culture of *Weissella cibaria* PEP23F and *Saccharomyces cerevisiae* AN6Y19. Compared to Raw-ABP and Chemically Acidified-ABP (CA-ABP), fermentation markedly increased the hydration properties of ABP. Fermentation led to the highest increases of total and insoluble dietary fibers (DF). Raw-, CA- and Fermented-ABP, at 5 and 10% (w w^–1^ of flour), were the ingredients for making fortified wheat breads. Addition of ABP and mainly fermentation enhanced dough water absorption and stability, and markedly increased the content of DF. Fortification mainly with 5% of Fermented-ABP did not interfere with bread rheology and color. As shown by profiling volatile compounds, Fermented-ABP imparted agreeable and specific sensory attributes, also appreciated by sensory analysis, and decreased bread hydrolysis index, and delayed mold contamination and firming. Fermented-ABP were suitable ingredients to fortify wheat bread formula, which agreed with bio-economy and environmental sustainability concepts.

## Introduction

The Codex Alimentarius classifies dietary fibers (DF) as polymers with 10 or more monomeric units, which are not hydrolyzable by human endogenous enzymes at the level of the small intestine. DF are naturally present in foods, extractable from edible raw materials or correspond to synthetic or modified polymers (Alba et al., 2019). Cellulose, β-glucan, hemicelluloses, pectin, inulin, resistant starch, and various oligosaccharides are the most common DF.

Nutrition guidelines from United States (Dietary Guidelines for Americans) and Europe (European Food Safety Authority Scientific Opinion) strongly recommend the daily intake of 25–30 g of DF. Consumers are aware of the benefits of dietary habits rich in DF, but the average daily intake of many populations/categories is still lower with respect to recommended doses (Stephen et al., 2017). Apart from foods, which naturally contain DF, a challenging industrial and dietary perspective considers food fortification to broaden the consumption. Under the bio-economy umbrella, and paying attention to environmental sustainability and waste recovery, the re-cycling of agriculture by-products naturally rich in DF conjugates creation of added economic value and food chain sustainability.

The industrial manufacturing of fruits and vegetables generates approximately 50% by-product wastes in the form of peels, cores, pomace, unripe and/or damaged fruits and vegetables (Padayachee et al., 2017). The most common use of these by-products is for animal feeding, processing into biogas or composting to obtain bio-fertilizers. Nevertheless, fruit and vegetable by-products and, in particular, apple by-products have high fiber content (e.g., up to 93.2 per 100 g of solids) (Nawirska and Kwasniewska, 2005), mainly consisting of insoluble fibers (cellulose and hemicellulose) and pectin (≥10%). Insoluble DF play a major role in bowel scouring, promotion of bowel health, and bulky stool development (Gómez and Martinez, 2017). On the other hand, soluble DF affect the gut microbiome functionality, predisposing to the synthesis of short-chain fatty acids with beneficial effects on the intestinal epithelium and gut immune system (Verspreet et al., 2016). DF also enhance weight management, lower blood cholesterol and control glycemic and insulin responses (Gómez and Martinez, 2017). Apples are among the most processed fruits worldwide, especially for juice and beverage-making (Rabetafikaa et al., 2014; Gómez and Martinez, 2017), and at European level, apples represent the fruit that generates the highest percentage of fruit waste (ca. 26%) (De Laurentiis et al., 2018). Thus, apples by-products represent a low-cost substrate available in large amounts, and the recovery of valuable components from the apple by-products may generate economical and environmental benefits. Apart DF, relevant amounts of phenolic compounds, magnesium (0.02–0.36 g per 100 g dry weight basis, equal to 8–150% RDI) and calcium (0.06–0.10 g per 100 g dry weight basis, equal to 6–10% RDI) are also available, depending on the type of apple by-product (Bhushan et al., 2008; Rabetafikaa et al., 2014; Gómez and Martinez, 2017). Phenolic compounds in apple by-products are mainly represented by flavonoids (catechin, epicatechin, procyanidin, phloridizin, phloretin-2′-xyloglucoside, quercetin, and quercetin glycosides) and hydroxycinnamic acid (chlorogenic acid). A plethora of biological activities have been attributed to the phenolic compounds (e.g., antioxidant, anti-inflammatory, antimicrobial, or cytotoxic properties), and several studies have correlated a diet rich in phenolics to a reduced incidence of chronic diseases (Costa et al., 2017). Renouncing this nutritional patrimony contravenes the basic and current rules of bio-economy and limits diet fortification.

Because of the positive influence of DF and other biogenic compounds during digestion of glycemic carbohydrates (e.g., starch), baked goods are ideal food systems to accommodate fruit and vegetable by-products. Most of these staple products have, indeed, rather high glycemic indexes, which somewhat prevent their consumption. Bread fortification with apple by-products combines waste re-cycling and dietary recommendations, and enlarge the market potential. Several fruit and vegetable by-products have been used for baked good fortification showing peculiar applications and results, which mainly depended on the species, part of the plant and industrial processing. Nevertheless, the addition of fruit and vegetable by-products in baked good formulas leads to losses of acceptability, because of the worsening of sensory and rheology attributes, and the variability of the nutritional value (Alba et al., 2019).

As one of the oldest and natural processes, lactic acid fermentation, mainly in form of sourdough process, has proven to enhance the acceptability and the nutritional value of leavened baked goods (Gobbetti et al., 2018). At the same time, literature has shown how lactic acid bacteria are part of the autochthonous population of fruits and vegetables and, how under controlled fermentation, they increase the bioavailability of DF, phenolic acids and/or enrich the matrices with biogenic compounds (Filannino et al., 2018).

This study aimed at using apple by-products to fortify wheat bread. Fortification (5 and 10%, w w^–1^ of flour) was with raw apple by-products, and with chemically acidified and fermented apple by-products, which latter underwent incubation for 48 h at 30°C. The characterization concerned hydration, biochemical, and nutritional attributes of apple by-products, and technology, shelf life sensory, and nutritional features of the fortified breads.

## Materials and Methods

### Apple By-Products

Apple by-products (ABP) were a mixture of pulp and peel residues (variety Imperatore), which resulted from filling during the manufacture of traditional sweet baked products. Frozen ABP were kindly supplied by the company PAN Surgelati Srl (Bolzano, Italy).

### Microorganisms and Culture Conditions

*Weissella cibaria* PEP23F, isolated from sweet pepper (Filannino, unpublished observations and genetic identification), *Leuconostoc mesenteroides* KI6, isolated from kiwifruit (Filannino et al., 2014; Filannino et al., 2016), *Lactobacillus plantarum* 3DM, isolated from sourdough (Corsetti et al., 2004), *Saccharomyces cerevisiae* AN6Y19 and *Hanseniaspora uvarum* AN8Y2C isolated from *Annona squamosa* (L.) fruits (Filannino, unpublished observations and genetic identification) were from the Culture Collection of the Department of Soil, Plant and Food Science, University of Bari Aldo Moro (Bari, Italy). Lactic acid bacteria strains identity was confirmed by partial sequencing of 16S rRNA and recA genes. Yeasts identity was established by sequencing the ITS1-5.8S-ITS2 region. All these species are frequently responsible for spontaneous fermentations of plant matrices and largely populate apple fruits (Valles et al., 2007; Savino et al., 2012). Lactic acid bacteria and yeast strains were maintained as stocks in 15% (vol vol^–1^) glycerol at −80°C and routinely propagated at 30°C for 24 h in MRS broth (Oxoid, Basingstoke, Hampshire, United Kingdom) and at 25°C for 24 h in Sabouraud Dextrose liquid medium (Oxoid), respectively. Previously, *W. cibaria* PEP23F showed the potential to synthesize exo-polysaccharides (Filannino, unpublished observations). In order to investigate this phenotypic trait, *W. cibaria* PEP23F was grown on MRS supplemented with 292 mM sucrose, 146 mM glucose and 146 mM fructose, and EPS were purified and quantified EPS as described by Minervini et al. (2010).

### Apple By-Products (APB) Fermentation

After thawing, ABP were blended with a vertical food processor (mod. R8 Robot Coupe, Bologna, Italy), heated at 90°C for 20 min, and cooled at 25°C before the inoculum. *W. cibaria* PEP23F, *L. mesenteroides* KI6, *L. plantarum* 3DM, *S. cerevisiae* AN6Y19 and *H. uvarum* AN8Y2C, were used singly or as binary starters, consisting of a yeast and a lactic acid bacterium. The cultivation of lactic acid bacteria and yeasts was on MRS at 30°C and SAB broths at 25°C, respectively, until reaching the late exponential phase of growth. Cells were harvested by centrifugation (10,000 × *g*, 10 min, 4°C), washed twice with 50 mM sterile potassium phosphate buffer (pH 7.0), and re-suspended in ABP at the final cell density of ca. 7.0 Log CFU g^–1^ (Xiao, 2006; Di Cagno et al., 2016). The fermentation was at 30°C up to 72 h. Sampling was before (Raw-ABP) and after fermentation (Fermented-ABP). ABP, without microbial inoculum but chemically acidified with lactic acid (final pH of ca. 3.5), were incubated under the same conditions and used as the control (CA-ABP). Microbial growth and acidification were monitored. A Foodtrode electrode (Hamilton, Bonaduz, Switzerland) measured the pH. Enumeration of lactic acid bacteria was on MRS agar (Oxoid), containing 0.1% of cycloheximide (Sigma-Aldrich, Steinheim, Germany), at 30°C for 48 h under anaerobiosis. Plate counting of yeasts was on Sabouraud Dextrose Agar, added of 150 ppm chloramphenicol, at 30°C for 72 h. Randomly amplified polymorphic DNA-PCR (RAPD-PCR) analysis was used to monitor selected cultures during fermentation. Lactic acid bacteria were biotyped through RAPD-PCR using P4, P7 and M13 primers, applying the same conditions previously described (Lhomme et al., 2015). Primers M13m and RP11 were used for typing of yeasts (Lhomme et al., 2015). RAPD-PCR profiles were acquired by the MCE-202 MultiNA microchip electrophoresis system (Shimadzu Italia s.r.l., Milan, Italy), using the DNA-2,500 (100 to 2,500 bp) kit according to the manufacturer’s instructions. Reproducibility of the RAPD profiles was assessed by comparing the PCR products obtained from DNA prepared from three independent cultures of the same isolate.

Based on growth and acidification data, the time of fermentation time was set at 48 h. Fermented-, Raw- and CA-ABP were freeze-dried, grounded and further analyzed.

### APB Hydration Properties

Water-holding capacity (WHC) was the quantity of water that was bound to the freeze-dried APB, without the application of any external force (except for gravity and atmospheric pressure). Accurately weighed dry sample (1 g) into a graduated test tube was added of 30 ml of water and hydration was allowed for 18 h. The supernatant was removed, the hydrated residue weight was recorded and drying at 105°C was lasting for 2 h to get the residual dry weight (Raghavendra et al., 2004). The calculation of WHC (g g^–1^) was according to the following equation:

WHC=(m-fm)d×m;d-1

where *m*_*f*_ is the weight (g) of the fresh residue and *m*_*d*_ is the weight (g) of the dry residue.

Water retention capacity (WRC) was the quantity of water that remained bound to the freeze-dried ABP samples following the application of an external force (pressure or centrifugation). The protocol and the equation were the same as those described above, except for centrifugation at 3,000 × *g* for 20 min (external force), which was applied after removal of the supernatant solution (Raghavendra et al., 2004).

Swelling capacity (SC) was the ratio between the sample volume when immersed in an excess of water and the sample weight prior hydration. Accurately weighed dry sample (0.2 g) was filled into a graduated test tube, 10 ml of water were added and hydration was allowed for 18 h. Subsequently, the volume attained by ABP was measured (Raghavendra et al., 2004). The calculation of SC (ml g^–1^) was according to the following equation:

SC=v×w;-1

where *v* is the sample volume (ml), and *w* is the sample weight (g) prior hydration.

### Biochemical Characterization of ABP

Carbohydrates (glucose and fructose), organic acids (lactic and acetic acids), and ethanol of ABP were determined through HPLC, using an ÄKTA Purifier system (GE Healthcare, Uppsala, Sweden), equipped with an Aminex HPX-87H ion exclusion column (Biorad) and Perkin Elmer 200a refractive index detector. Sugars, organic acids, and ethanol used as the standards were purchased from Sigma-Aldrich. Total titratable acidity (TTA) was measured on 10 g of freeze-dried ABP, homogenized with 90 ml of distilled water (Classic Blender, PBI International), and expressed as the amount (ml) of 0.1M NaOH to get the value of pH of 8.3.

The enzymatic-gravimetric AOAC Official Method 991.43 was used to determine the total soluble and insoluble dietary fibers of freeze-dried ABP. According to the protocol, dried samples were suspended in MES-TRIS buffer (pH 8.2), and treated with amylase, protease and amyloglucosidase at the respective values of pH. After enzymatic digestion, the insoluble dietary fibers were separated by filtration, washed with ethanol and acetone, further dried and weighed. Soluble dietary fibers were precipitated by ethanol, filtered, washed with ethanol and acetone, further dried and weighed. Soluble and insoluble fibers were also incinerated and weighed to determine the ash value. Freeze-dried samples of ABP were also subjected to the determination of the protein content by AOAC official methods 978.02. The concentration of both insoluble and soluble fibers was calculated subtracting the values of ash and insoluble protein from the dried weight of residues, and it was expressed as the percentage ratio between fiber weight and sample weight. The total dietary fiber was the sum of soluble and insoluble fibers.

### Bile Acid Retardation Index

Bile acid retardation retardation index (BRI) was used to monitor the effect of fiber on cholesterol metabolism. Bile acids are synthesized from cholesterol and are released from the gall bladder in response to food intake. Most bile acids are reabsorbed from the gut and only a small fraction is excreted in urine and feces. However, bile acids binding prevent their reabsorption and facilitates their excretion (Adiotomre et al., 1990). To assay the BRI a glycocholic acid solution was prepared by mixing phosphate buffer at pH 7 with 1 g l^–1^ of sodium azide and 15 mmol l^–1^ of glycocholic acid. Freeze-dried ABP (0.2 g) was mixed with 15 ml of glycocholic acid solution and transferred into a dialysis membrane (cut-off 14,000) (Sigma-Aldrich). The dialysis bag was placed into 100 ml of phosphate buffer with 1 g l^–1^ sodium azide (pH 7) at 37°C. Glycocholic acid without freeze-dried ABP was used as the control. After 60 min, an aliquot of the dialyzate (2 ml) was removed for analysis, and the content of glycocholic acid was measured through HPLC, using an ÄKTA Purifier system (GE Healthcare), equipped with an XTerra MS C18 column (Waters, Brussels, Belgium) and an UV detector at 200 nm. The mobile phase consisted of methanol-0.01M potassium phosphate (monobasic) 130:70 (v v^–1^) pH 5.75, and elution was at a flow rate of 0.2 ml min^–1^. BRI was calculated according the following equation:

BRI(%)=100-[(Cd×100)×Cc]-1;

where, *Cd* was the total glycocholic acid diffused from ABP samples (mmol l^–1^) and *Cc* was the total glycocholic acid diffused from the control (mmol l^–1^).

### Bread-Making and Technological Analyses

Breads, containing Raw-, Fermented- or CA-ABP at 5 and 10% (w w^–1^ of flour) and having a dough yield (DY) of 200, were manufactured at the pilot plant of the Department of Soil, Plant and Food Science of the University of Bari (Italy). According to DY, the formula was 50.0% (w w^–1^) of white wheat (*Triticum aestivum*) flour (type 00) fortified with freeze-dried ABP and 50.0% (w w^–1^) of tap water. Baker’s yeast was added at the percentage of 1.5% (w w^–1^). A baker’s yeast wheat bread (BY) was manufactured without the addition of ABP (DY of 200) and used as the control. The gross composition of flour was as follows: moisture 13.19%, protein 9.9% of dry matter (d.m.), ash 0.81% of d.m., total carbohydrates 75.5% of d.m.; fat 1.3% of d.m. and dietary fiber 1.5% of d.m. Doughs were mixed at 60 g for 5 min with an IM 5-8 high-speed mixer (Mecnosud, Flumeri, Italy) and fermented at 30°C for 2 h. All breads were baked at 220°C for 30 min (Combo 3, Zucchelli, Verona, Italy).

Degree of softening and stability of doughs were estimated by a Brabender farinograph (Brabender OHG Duisburg, Germany), according the manufacturer instructions. The difference between the starting (S) and final (F) resistance values, expressed in Farinograph Units (FU), was considered as a measure of the degree of dough softening. The stability was defined as the time between the first and second intersecting point of the upper trace of the torque curve with the consistency line.

The specific volume of breads was measured using the rapeseed displacement method (ACCI Method 10-05.01, Guidelines for Measurement of Volume by Rapeseed Displacement). In detail, bread loaf was weighed and placed into a 2 L beaker. After having completely covered the bread by pouring rapeseeds in the beaker, the volume was measured (cm^3^). Then, the bread was removed from the beaker and the volume of rapeseeds alone was measured. Specific volume (cm^3^ g^–1^) was calculated as the difference between the two measured volumes, divided by bread weight. The crumb cells of breads were analyzed after 24 h of storage using the image analysis technology. Images of the sliced breads were captured using an Image Scanner (Amersham Pharmacia Biotech, Uppsala, Sweden). Images were scanned full-scale at 300 dots per inch and analyzed in gray scale (0–255). Image analysis was performed using the UTHSCSA ImageTool program (Version 2.0, University of Texas Health Science Centre, San Antonio, TX, United States available by anonymous FTP from maxrad6.uthscsa.edu). A threshold method was used for differentiating gas cells and non-cells (Crowley et al., 2000). Analysis was carried out on two sub-images (1001 × 1393 pixels, field of view) selected within the bread slice. The crumb cell features recovered were number, area, perimeter, and gas cell to total area ratio. Color was measured in three different points of bread crust using a Minolta CR-10 Camera. The *L*, *a*, *b* color space analysis method was used, where *L* represents lightness (white-black) and *a* and *b* the chromaticity co-ordinates (red-green and yellow-blue, respectively). Results were reported in the form of a color difference, $d\,E_{a\,b}^{*}$, as follows:

$$d\,E_{a\,b}^{*}=\sqrt{{\left(d\,L\right)}^{2}+{\left(d\,a\right)}^{2}+{\left(d\,b\right)}^{2}}$$

where *dL*, *da* and *db* are the differences for *L*, *a*, and *b* values between sample and reference (a white ceramic plate having *L* = 93.4, *a* = 1.8, and *b* = 4.4).

### Determination of Volatile Compounds

Volatile compounds (VOCs) were extracted by HS-SPME technique using a DVB-CAR-PDMS fiber (Supelco, Bellefonte, PA, United States) according to Gambacorta et al. (2019), with some modifications. Bread sample (1 g) and 10 μL of 2-octanol (internal standard, 81.9 ng l^–1^ in water) were placed in 20 ml screw-cap vial, tightly capped with a PTFE-silicon septum, and conditioned for 10 min at 40°C. A TriPlus RSH^TM^ Autosampler (Thermo Fisher Scientific, Rodano, Italy) was used to optimize and standardize the entire extraction procedure. The fiber then was introduced into the headspace of the vial for 40 min at 40°C. Then, desorption of volatiles from fiber took place in a spitless mode for 3 min at 220°C. The separation of VOCs was performed by a Trace 1300 gas chromatograph (Thermo Fisher Scientific), equipped with a VF-WAXms capillary column (Agilent, Santa Clara, CA, United States), 60 m length × 0.25 mm I.D. × 0.25 μm film, and coupled with an ISQ single quadrupole mass spectrometer (Thermo Fisher Scientific). The chromatographic conditions were: oven, 45°C (5 min) to 210°C at 4°C/min, held for 3 min; detector, source temperature 250°C; transfer line temperature 250°C; carrier gas, helium at constant flow of 0.4 mL min^–1^. The impact energy was 70 eV. Data were acquired using the full-scan mode in the range of 35 to 150 m/z at an acquisition rate of 7.2 Hz. VOCs were tentatively identified by comparing the experimental spectra with those reported in the NIST Library and with those obtained by the available pure standard compounds. Volatiles were quantified using relative areas related the 2-octanol as internal standard. Acquisition and processing of peaks was carried out using Xcalibur v 4.1 software (Thermo Fisher Scientific).

### *In vitro* Starch Hydrolysis

The analysis of starch hydrolysis simulates the *in vivo* digestion of starch. Bread portions, contained 1 g of starch (determined in bread), were given in randomized order to 10 volunteers. The analysis was performed as described by Liljeberg et al. (1996). The K-FRUGL 09/13 kit (Megazyme, Intl., Ireland) was used for the determination of the glucose concentration. The degree of starch digestion at different times (0–180 min, at 30-min intervals) was expressed as the ratio of hydrolyzed starch. Kinetics of hydrolysis was elaborated using the statistical software Statistica 7.0 (Statsoft) according a first order equation: *C* = *C*_8_ (1– e^–^*^*kt*^*) where *C* is the concentration at *t* time, *C*_8_ is the equilibrium concentration, k is the kinetic constant and t is the chosen time (De Angelis et al., 2009). Baker’s yeast wheat bread (BY) was used as the reference (HI = 100).

### Staling Rate

The staling rate was assessed by determining both crumb and crust moisture content and water activity (a_w_) of baked bread after cooling, and bread stored in perforated plastic bags at room temperature (25°C) after 1, 2, and 5 days from manufacture. Moisture was determined according to the ICC-Standard method 110/1 (1976), whereas a_w_ was measured with an Aqua Lab apparatus (Aqua Lab, Model 3TE, Decagon Devices, Inc., Pullman, WA, United States).

### Microbial Stability

After cutting, bread slices (12 and 1.5 cm, height and width, respectively) underwent packaging on polyethylene bags, to maintain constant moisture, and incubation at room temperature lasted 14 days. The assessment of the environmental mold contamination was by visual observation of the slices, with the approximate quantification as the percentage of the surface covered by visible mold mycelia.

### Sensory Analysis

After 24 h from baking, a laboratory panel assessed the sensory attributes of freeze-dried ABP fortification. The panel of judges consisted of 10 researchers with multi-year bread-tasting experience. Three introductory sensory training sessions were carried out for discussing the sensory attributes with the panelists. Before sensory evaluation, breads were cut into 1.5 cm thick slices. Slices were cut into four pieces and served at room temperature, under normal (daylight) illumination. Each bread, identified by a code number, was given to each panelist on a single tray. Samples were served randomly. Sensory attributes were: crust darkness, crumb darkness, elasticity of crumb, crumb softness, crust crispness, crumb dryness, sourness, bitterness, rancid, astringency, toasted, nutty, fruitiness, sweetness, fermented, aroma intensity. Each sensory attribute was rated with a score from 0 (lowest) to 10 (highest).

### Statistical Analysis

For each condition, samples obtained from three independent experiments were analyzed in triplicate. Analysis was performed using analysis of variance (ANOVA) test for multiple comparisons (one-way ANOVA followed by Tukey’s procedure at *P* < 0.05), using the statistical software, Statistica 7.0 (Statsoft). VOCs (ppm) and sensory attributes were analyzed by principal component analysis (PCA), using a covariance matrix with the software Statistica 7.0 (Dijksterhuis, 1997).

## Results

### Fermentation

A panel of lactic acid bacteria and yeasts, which commonly populate and ferment apple fruits, were selected to ferment ABP. *W. cibaria* PEP23F was included into the panel as it showed the potential to synthesize exo-polysaccharides up to 8.4 g kg^–1^ in MRS supplemented with sucrose, glucose, and fructose.

Lactic acid bacteria and yeasts were cultivated on ABP as single and binary cultures (Supplementary Figure S1). The initial cell density was always ca. 7 Log CFU g^–1^. Raw-ABP and chemically acidified ABP also subjected to incubation but without inoculum (CA-ABP) were the controls. From the initial value of ca. 7 Log CFU g^–1^, the presumptive cell number of lactic acid bacteria further increased by 1.92 ± 0.26, 1.25 ± 0.24, and 1.93 ± 0.18 Log CFU g^–1^ after 48 h of ABP fermentation with single cultures of *L. mesenteroides* KI6, *W. cibaria* PEP23F, and *L. plantarum* 3DM, respectively (Supplementary Figure S1). After the same time of fermentation, the single culture of *S. cerevisiae* AN6Y19 increased by ca. 1 log cycle. On the contrary, *H. uvarum* AN8Y2C decreased by ca. 2 log cycles with respect to the initial inoculum. When used binary cultures of lactic acid bacteria and *S. cerevisiae* AN6Y19, bacteria increased during 24 h (ca. 1.5 log cycle), then decreased at 48 h to the final cell density of 7.9 ± 0.34 (*L. mesenteroides* KI6), 7.51 ± 0.32 (*W. cibaria* PEP23F), and 7.77 ± 0.24 (*L. plantarum* 3DM) Log CFU g^–1^ (Supplementary Figure S1). *S. cerevisiae* AN6Y19 showed an increase of ca. 1 log cycle. Cultivable cells of *H. uvarum* AN8Y2C also decreased under co-cultivation with lactic acid bacteria. Similarly to co-cultivation with *S. cerevisiae* AN6Y19, binary cultures of lactic acid bacteria and *H. uvarum* AN8Y2C allowed a bacterial increase during 24 h (ca. 1.5 log cycle), followed by a decrease at 48 h to the final density of 7.54 ± 0.24 (*L. mesenteroides* KI6), 7.9 ± 0.26 (*W. cibaria* PEP23F) and 8.61 ± 0.26 (*L. plantarum* 3DM) Log CFU g^–1^. Presumptive lactic acid bacteria were also detectable in Raw-ABP (3.32 ± 0.26 Log CFU g^–1^) and CA-ABP (2.42 ± 0.24 Log CFU g^–1^) before incubation. After 48 h of fermentation, their cell number increased by ca. 4 log cycles in CA-ABP. Similarly, yeasts were initially present in Raw-ABP (4.1 ± 0.21 Log CFU g^–1^) and in CA-ABP (2.9 ± 0.25 Log CFU g^–1^), showing an increase of ca. 2.5 log cycles after incubation (Supplementary Figure S1).

RAPD-PCR analysis monitored selected cultures during fermentation. Except for *H. uvarum* AN8Y2C, which disappeared after 48 h when co-inoculated with L. *plantarum* 3DM, all the other strains were detectable after 48 h of fermentation at 30°C (Supplementary Figure S2).

### ABP Hydration Properties

**FIGURE 1:**
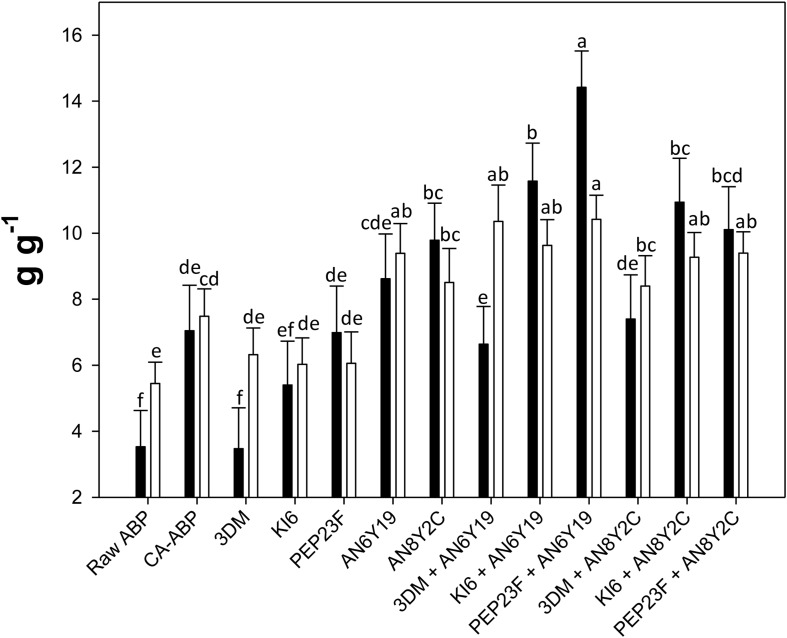
Water-holding capacity (WHC) (black bars) and water retention capacity (WRC) (white bars) of raw apple by-products (Raw-ABP), and chemically acidified ABP (CA-ABP) and Fermented-ABP, which were incubated for 48 h at 30°C. Fermentation (Fermented-ABP) was with selected single and binary cultures of *Weissella cibaria* PEP23F, *Leuconostoc mesenteroides* KI6, *Lactobacillus plantarum* 3DM, *Saccharomyces cerevisiae* AN6Y19 and *Hanseniaspora uvarum* AN8Y2C. Data are the means (± SD) of three independent experiments analyzed in triplicate. Data were subjected to one-way ANOVA followed by Tukey’s procedure at *P* < 0.05. Bars with different superscript letters differ significantly (*P* < 0.05).

Water holding capacity and WRC of Raw-ABP were 3.53 ± 1.1 and 5.45 ± 0.6 g g^–1^, respectively (Figure 1). Because of the chemical acidification, CA-ABP showed a consistent increase of both these values (7.04 ± 1.3 and 7.48 ± 0.8 g g^–1^, respectively). Overall, the fermentation further increased (*P* < 0.05) WHC and WRC. Single cultures of *S. cerevisiae* AN6Y19 and *H. uvarum* AN8Y2C led to WHC of 8.62 ± 1.4 and 9.79 ± 1.12 g g^–1^, and WRC of 9.39 ± 0.9 and 8.51 ± 1.03 g g^–1^, respectively. Fermentation of ABP with the binary cultures of yeasts and lactic acid bacteria further enhanced WHC and WRC. The highest (*P* < 0.05) values were for Fermented-ABP with the binary culture of *W. cibaria* PEP23F and *S. cerevisiae* AN6Y19 (14.4 ± 1.1 and 10.4 ± 0.7 g g^–1^, respectively).

Compared to Raw-ABP and CA-ABP, the fermentation with selected strains did not allow significant (*P* < 0.05) variations of the swelling capacity (SC) (15.5–18.0 ml g^–1^).

In conclusion, growth data and hydration properties oriented toward the selection of the binary culture between *W. cibaria* PEP23F and *S. cerevisiae* AN6Y19. It underwent further characterization, and using for bread making.

### ABP Physical–Chemical Features and Bile Acid Retardation Index

Raw-ABP had the value of pH of 4.07 ± 0.09 (Table 1). The value of pH of Fermented-ABP (3.85 ± 0.09) was almost comparable with that of CA-ABP. As expected, TTA was significantly (*P* < 0.05) the highest for Fermented-ABP and CA-ABP. Glucose and fructose were the dominant carbohydrates of Raw-ABP (19.3 ± 1.4 and 35.4 ± 2.2 g kg^–1^ dry weight, respectively). Fermented-ABP showed a substantial (*P* < 0.05) decrease of glucose and fructose (ca. 80% w/w for both carbohydrates). A slight but significant (*P* < 0.05) decrease occurred for CA-ABP. Main fermentation metabolites were lactic (2.77 ± 0.24 g kg^–1^ dry weight) and acetic (2.95 ± 0.32 g kg^–1^ dry weight) acids (Table 1). Although the use of *S. cerevisiae* AN6Y19 and/or the presence of autochthonous yeasts, ethanol was not detectable in any samples. Presumably, this was because of the freeze-dried treatment before analysis. Raw-ABP had a content of total DF of 44.9 ± 2.0% dry weight, consisting of insoluble (38.2 ± 1.9% dry weight) and soluble (6.7 ± 0.8% dry weight) DF (Table 1). Fermentation of ABP favored the highest (*P* < 0.05) percentage increases of total (40%) and insoluble (44%) DF, and a significant percentage increase of soluble DF (19%). On the contrary, the increase observed with CA-ABP was weak, with the exception of soluble DF (52%). Freeze-dried ABP samples underwent to measurements of bile acid retardation. After 1 h of dialysis, the retention of glycocholic acid from Fermented-ABP was markedly the highest (39.2 ± 0.2%) with respect to Raw-ABP (8.4 ± 0.3%) and CA-ABP (18.7 ± 0.3%).

**TABLE 1:** Physical and chemical characteristics of raw apple by-products (Raw-ABP), and chemically acidified ABP (CA-ABP), and Fermented-ABP incubated at 30°C for 48 h.

	**Raw-ABP**	**CA-ABP**	**Fermented-ABP**
pH	4.07 ± 0.09^a^	3.82 ± 0.10^b^	3.85 ± 0.09^ab^
TTA (mL 0.1M NaOH 10 g^–1^ dry weight)	82.0 ± 0.7^c^	125.0 ± 1.9^a^	117.2 ± 1.6^b^
Glucose (g kg^–1^ dry weight)	19.26 ± 1.4^a^	15.43 ± 1.8^b^	3.77 ± 0.4^c^
Fructose (g kg^–1^ dry weight)	35.38 ± 2.2^a^	28.79 ± 2.7^b^	6.93 ± 0.8^c^
Lactic acid (g kg^–1^ dry weight)	0.65 ± 0.26^b^	2.60 ± 0.21^a^	2.77 ± 0.24^a^
Acetic acid (g kg^–1^ dry weight)	1.41 ± 0.21^b^	1.30 ± 0.32^a^	2.95 ± 0.32^a^
Ethanol (g kg^–1^ dry weight)	*n*.*d*.	*n*.*d*.	*n*.*d*.
Soluble dietary fibers (% dry weight)	6.7 ± 0.8^b^	10.2 ± 0.5^a^	8.0 ± 0.7^b^
Insoluble dietary fibers (% dry weight)	38.2 ± 1.9^c^	41.8 ± 2.9^b^	55.0 ± 1.6^a^
Total dietary fibers (% dry weight)	44.9 ± 2.0^c^	52.0 ± 3.1^b^	63.0 ± 2.4^a^

*Fermentation (Fermented-ABP) was with the selected binary culture of *Weissella cibaria* PEP23F and *Saccharomyces cerevisiae* AN6Y19. Data are the means (± SD) of three independent experiments analyzed in triplicate. Data were subjected to one-way ANOVA followed by Tukey’s procedure at *P* < 0.05. ^*a*–*c*^Means within the row with different letters are significantly different (*P* < 0.05). n.d., not detected.*

### Technology Features of Bread Making With ABP

Table 2 shows the degree of softening and stability of doughs fortified with Raw-, CA- and Fermented-ABP at concentrations of 5 and 10% (w w^–1^ of flour). Both the addition of ABP and fermentation influenced the water absorption, and, especially, the stability and degree of softening of doughs. Regarding the dose-effect relationship, dough stability positively correlated with the amount of Raw-ABP added, whereas the effect of fermentation (Fermented- or CA-ABP) was independent of the addition. The degree of softening seemed to correlate positively with the amount of Fermented- or CA-ABP added. Compared to Control dough (wheat flour and water), the addition of Raw-ABP slightly but significantly (*P* < 0.05) affected dough rheology. The best effect (*P* < 0.05) on water absorption and dough rheology was with Fermented-ABP. It led to the highest percentage increases of water absorption (ca. 4%) and stability (ca. 213%), and the lowest percentage decreases of the degree of softening 10 min after starting the test (64–73%), and 12 min after development time (36–47%). High water absorption combined with high stability and low degree of softening indicated a flour of optimal quality. CA-ABP caused similar changes, but it determined a lower (*P* < 0.05) water absorption capacity and a higher (*P* < 0.05) degree of softening than Fermented-ABP.

**TABLE 2:** Water absorption and kneading properties of wheat flour fortified with apple by-products (ABP) as estimated by a Brabender farinograph (Brabender OHG, Duisburg, Germany).

	**Water absorption at 500 FU (%)**	**Stability (min)**	**Degree of softening [10 min after test start] (FU)**	**Degree of softening [12 min after development time–ICC standard] (FU)**
Control dough	59.2 ± 0.3^b^	2.9 ± 0.1^c^	67 ± 1.0^a^	80 ± 1.1^a^
Raw-ABP (5% w/w of flour)	56.4 ± 0.4^c^	2.5 ± 0.1^d^	57 ± 1.2^b^	79 ± 1.2^a^
Raw-ABP (10% w/w of flour)	56.2 ± 0.2^c^	7.4 ± 0.2^b^	54 ± 0.8^c^	79 ± 1.2^a^
CA-ABP (5% w/w of flour)	58.8 ± 0.6^b^	8.9 ± 0.2^a^	23 ± 1.1^d^	48 ± 1.0^d^
CA-ABP (10% w/w of flour)	59.8 ± 0.3^b^	8.8 ± 0.1^a^	25 ± 0.8^d^	62 ± 1.9^b^
Fermented-ABP (5% w/w of flour)	59.6 ± 0.3^b^	9.1 ± 0.2^a^	18 ± 1.9^e^	42 ± 1.8^e^
Fermented-APB (10% w/w of flour)	61.5 ± 0.4^a^	9.0 ± 0.3^a^	24 ± 0.9^d^	51 ± 0.7^c^

*Raw ABP (Raw-ABP), and chemically acidified ABP (CA-ABP) and Fermented-ABP, which were previously incubated at 30°C for 48 h. Fermentation (Fermented-ABP) was with the selected binary culture of *Weissella cibaria* PEP23F and *Saccharomyces cerevisiae* AN6Y19. Raw-ABP, CA- and Fermented-ABP were added at concentrations of 5 and 10% (w w^–1^ of flour). Dough manufactured without the addition of ABP was the control (Control dough). Data are the means (± SD) of three independent experiments analyzed in triplicate. Data were subjected to one-way ANOVA followed by Tukey’s procedure at *P* < 0.05. ^*a*–*e*^Means within the column with different letters are significantly different (*P* < 0.05).*

After baking, BY had the highest specific volume (Supplementary Table S1). As expected, ABP, especially when added at 10%, slightly decreased the specific volume. Nevertheless, fortification with 5% of Fermented-ABP did not interfere with the specific volume. The crust color (dE) of both Fermented-ABP breads did not significantly (*P* > 0.05) vary compared to BY bread (Supplementary Table S1). Digital images underwent pre-processing to detect crumb cell-total area by a binary conversion (black/white pixels) (Supplementary Table S1). Gas cell-total area (corresponding to the black pixel ratio) of Fermented-ABP breads was significantly (*P* < 0.05) lower than those of BY and Raw- and CA-ABP breads. Nevertheless, breads fortified with Fermented-ABP, especially at 5%, showed rounder and uniform crumb cells. BY, Raw- and CA-ABP breads had more uneven crumb cell distributions (Supplementary Figure S3).

### Volatile Compounds

**FIGURE 2:**
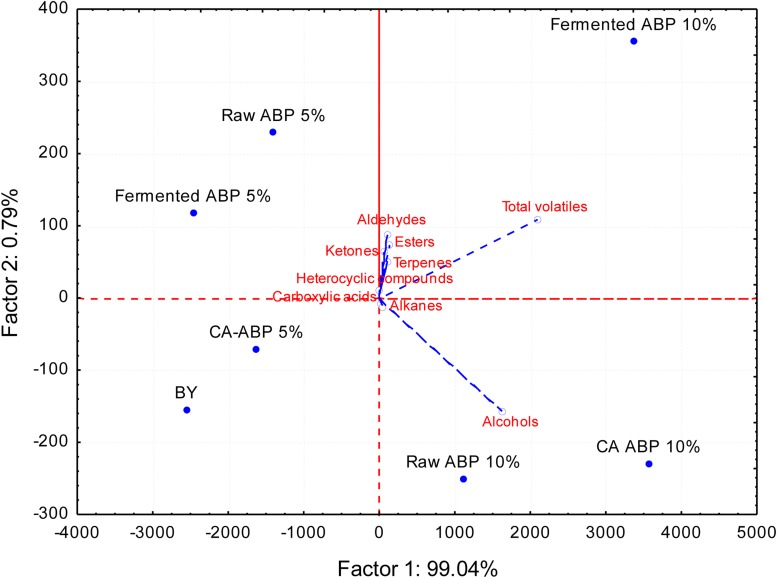
Principal component analysis (PCA) biplot based on volatile compounds (ppm) data of bread manufactured with wheat flour fortified with apple by-products (ABP):Raw ABP (raw-ABP), and chemically acidified ABP (CA-ABP) and Fermented-ABP, which were previously incubated at 30°C for 48 h. Fermentation (ABP-fermented) was with selected binary culture ofy *W. cibaria* PEP23F and *S. cerevisiae* AN6Y19. Raw-, CA-, and Fermented-ABP were added at concentrations of 5 and 10% (w w^–1^ of flour). Baker’s yeast wheat bread (BY), manufactured without addition of ABP, was the control.

Eighty-one VOCs were identified, and grouped according to the following chemical classes: alcohols (19 compounds identified), esters (14), aldehydes (14), ketones (12), carboxylic acids (8), heterocyclic compounds (10), terpenes (2), and alkanes (2) (Supplementary Table S2). At a glance, the level of VOCs differentiated the breads (Figure 2 and Supplementary Table S2). Compared BY bread, the addition of ABP significantly (*P* < 0.05) increased the amount of VOCs. The increase was proportional to fortification (5 and 10%, w w^–1^ of flour). The highest levels of VOCs were found in bread added of 10% (w w^–1^ of flour) of Fermented-ABP (7676 ± 778 ppm), and CA-ABP (7572 ± 94 ppm), followed by Raw-ABP (5628 ± 446 ppm). Alcohols were the VOCs most abundant, with ethanol, isoamyl alcohol, and phenylethyl alcohol at the highest levels (*P* < 0.05). Aldehydes (*n*-pentanal, *n*-heptanal, octanal), esters (isoamyl acetate, butanoic acid, 2- methyl-,hexyl ester, ethyl caprylate), and ketones (2-octanone, 1-octen-3-one, 6-Methyl-5-hepten-2-one) also markedly contribute to the VOCs profile of breads fortified with ABP, especially when 10% (w w^–1^ of flour) of Fermented-ABP was added.

### *In vitro* Starch Hydrolysis

The rate of *in vitro* starch hydrolysis is a presumptive measure of the glycemic index (GI) of healthy subjects (Åkerberg et al., 1998). The value of hydrolysis index (HI) of breads fortified with Fermented-ABP at 5 and 10% (w w^–1^ of flour) was 92 ± 1 and 81 ± 2%, respectively. These values were lower compared to those found in breads with Raw-ABP at 5 and 10% (w w^–1^ of flour) (98 ± 1 and 94 ± 1%, respectively) and CA-ABP (94 ± 1 and 88 ± 2%, respectively). BY bread had a HI of 100 ± 1%.

### Staling, Microbial Stability, and Sensory Analysis

**FIGURE 3:**
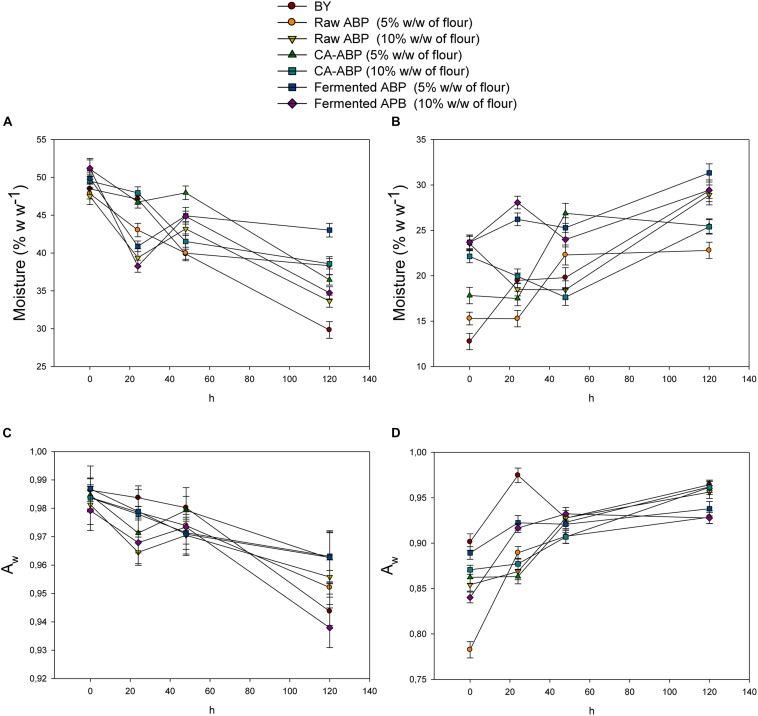
Moisture and a_w_ of crumb **(A,C)** and crust **(B,D)** in bread manufactured with wheat flour fortified with apple by-products (ABP):Raw ABP (raw-ABP), and chemically acidified ABP (CA-ABP) and Fermented-ABP, which were previously incubated at 30°C for 48 h. Fermentation (ABP-fermented) was with selected binary culture of *W. cibaria* PEP23F and *S. cerevisiae* AN6Y19. Raw-, CA-, and Fermented-ABP were added at concentrations of 5 and 10% (w w^–1^ of flour). Baker’s yeast wheat bread (BY), manufactured without addition of ABP, was the control.

The rate of firming (staling) is a function of bread (including crust) moisture content. As the bread moisture decreases, the rate of firming increases. As expected, all breads during 5 days of storage showed a clear and well-defined trend for crumb and crust moistures, which decreased and increased, respectively (Figures 3A,B). Nevertheless, the rate of crumb moisture loss significantly (*P* < 0.05) differed among breads. After 5 days of storage, the bread fortified with 5% of Fermented-ABP showed the highest value of crumb moisture. Although, the values of Aw for crumb and crust behaved almost similarly to the moisture content, the effects of ABP on this parameter were not statistically relevant (*P* > 0.05) (Figures 3C,D).

After baking, bread slices underwent cutting, packaging in polyethylene bags and storage at room temperature. Monitoring for mold contamination lasted 14 days (Supplementary Table S3). Before 11 days of storage, the contamination did not occur. It became evident at day-12, where the slice surfaces of all breads had an almost similar level of fungal growth, which ranged from 1 to 5%. The mold contamination markedly increased at 14 days of storage, except for slices from breads fortified with 5 and, especially, 10% of Fermented-ABP. This latter still had a mold contamination comparable to that found after 11 days of storage.

**FIGURE 4:**
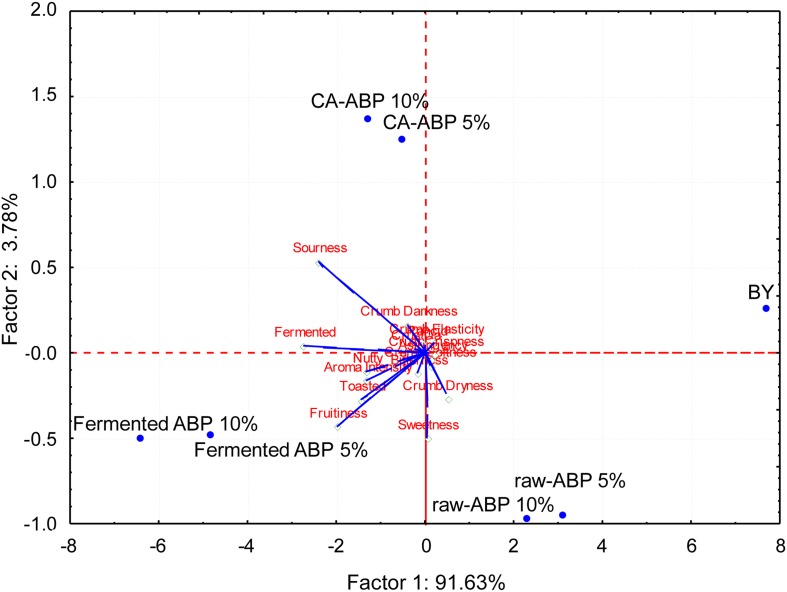
Principal component analysis biplot based on sensory analysis data of bread manufactured with wheat flours fortified with apple by-products (ABP):Raw ABP (raw-ABP), and chemically acidified ABP (CA-ABP) and ABP-fermented, which were previously incubated at 30°C for 48 h. Fermentation (ABP-fermented) was with the selected binary culture of *W. cibaria* PEP23F and *S. cerevisiae* AN6Y19. Raw-, CA-, and Fermented-ABP were added at concentrations of 5 and 10% (w w^–1^ of flour). Baker’s yeast wheat bread (BY), manufactured without addition of ABP, was the control. Sensory attributes were: crust darkness, crumb darkness, crumb elasticity, crumb softness, crust crispness, crumb dryness, sourness, bitterness, rancid, astringency, toasted, nutty, fruitiness, sweetness, fermented, aroma intensity.

Aiming at elucidating the effect of ABP fortification and fermentation on bread sensory attributes, BY, and Raw-, CA-, and Fermented-ABP were subjected to sensory analysis. PCA, representing 94% of the total variance of the data (Figure 4), showed that BY clearly separated from breads fortified with ABP. This was mainly due to the lowest scores received for sourness, fermented, fruitiness, nutty, and toasted attributes, and aroma intensity. Raw-ABP breads were perceived mainly for sweetness and crumb dryness attributes. CA-ABP breads scattered on the opposite and upper part of the plane, being characterized for crumb darkness and, especially, sourness. Breads manufactured with the addition of 5 and 10% Fermented-ABP scattered on the left zone of the plane because sharing the highest scores for fermented, fruitiness, sourness, toasted and nutty attributes, and aroma intensity.

## Discussion

Fruit and vegetable by-products deserve interest because inexpensive, available in large amount, nutritionally rich, and because their disposal entails an environmental impact and significant expenses. Indeed, food fortification with fruit and vegetable by-products has already addressed the interest toward numerous plant species such as banana, apple, grape, citrus fruits, and berry (Rabetafikaa et al., 2014; Gómez and Martinez, 2017). In principle, baked goods are suitable staple foods to be fortified, but the use of these unconventional ingredients caused declining technology and sensory attributes since the relevant increase of DF content and side effects by other chemical compounds. High levels of supplementation with dietary fiber lead to weaken the protein matrix with the consequent reduction of loaf volume, increased crumb firmness and darkening of crumb appearance (Grigor et al., 2016). Lastly, the ratio between soluble and insoluble forms of DF, and their nutritional availability and physiological activity depend on the characteristics of the raw by-products and tailored bakery processes (Verspreet et al., 2016).

Innovatively, this study proposed the preliminary fermentation of ABP with a selected binary culture of *W. cibaria* PEP23F and *S. cerevisiae* AN6Y19, and the subsequent use for manufacturing fortified wheat bread. Although the composition and functional properties of vegetable and fruit by-products depend on variables, such as origin, ripening degree, thermal and enzyme (pectinases) treatments (Rabetafikaa et al., 2014), these raw materials are highly prone to microbial growth (Russ and Meyer-Pittroff, 2004). Indeed, *W. cibaria* PEP23F and *S. cerevisiae* AN6Y19 grew and favored an increase of the hydration properties compared to Raw-ABP. The fermentation process consisted of two main steps, where the first one was dominated by *W. cibaria* PEP23F and the second one by *S. cerevisiae* AN6Y19. This sequence was probably due to the interaction between lactic bacteria and yeasts. Several studies have shown that the production of inhibitory metabolites by yeasts, such as ethanol, medium chain fatty acids, and antibacterial peptides or proteins, may have an inhibitory effect on lactic acid bacteria during plant matrices fermentation (Lonvaud-Funel et al., 1988; Dick et al., 1992; Capucho and San Romao, 1994; Vailiant et al., 1995; Mendoza et al., 2010). This antagonistic effect likely led the yeast to dominate during the second fermentation step. Anyway this succession allowed both to PEP23F and AN6Y19 to grow and to modify the DF composition of ABP. Hydration properties of DF depend on particle size and void spaces, fibrous or non-fibrous nature, solubility, chemical configuration and structure, and presence or absence of hydrophilic sites. As the fermentation clearly affected DF composition of ABP, it increased water uptake by ABP because the structure rupture of insoluble fibers, which combined the occurrence of hydroxyl groups, large porosity and high surface area (Chen et al., 2019). *S. cerevisiae* synthesizes extracellular enzymes that directly react with plant cell wall (Zhang et al., 2017), and the synthesis of exo-polysaccharides by *W. cibaria* PEP23F might have increased to the water binding capability of the system (Zannini et al., 2018). The enzymatic activity of *S. cerevisiae* AN6Y19, affecting the structure of insoluble fibers, combined to release of EPS by *W. cibaria* PEP23F likely maximized the hydration properties of ABP.

Besides the well-known effects on nutrient absorption and prebiotic activity, DF consumption has been correlated with a low risk of developing colon cancer, and may contribute to the reduction of cholesterol level. Compared to Raw-ABP, fermentation increased the bile acid binding activity with an effect comparable that found for other potent berry by-products (Lim et al., 2018). DF, in particular pectin (Brown et al., 1999), bind bile acids, and prevent their reabsorption, thus facilitating the excretion. Interactions between DF and bile acids at the level of the small intestine result in a higher excretion of bile acids, which increases the hepatic synthesis of bile acids from blood cholesterol.

Apple by-products at 5 and 10% (w w^–1^ of flour) were the ingredients to fortify wheat bread. No previous studies, with almost the same approach, exceeded the fortification with 10% of fruit or vegetable by-products and, in general, at these concentrations a loss of bread acceptability became evident (Gómez and Martinez, 2017). The manufacture of fortified baked goods without compromising their sensory appeal is the industrial challenge. Consumers do not like constraints; usually they rank nutrition beyond acceptability (Sandvik et al., 2018). It is precisely the increased content of DF, which renders breads with poor specific volume, softness and taste acceptability (Wang et al., 2002). Lower volume and higher hardness of breads depend on the decreased gas holding capacity of the dough because the dilution of the gluten content with the partial substitution with fruit or vegetable by-products (Gómez et al., 2003; Rosell and Santos, 2010). Compared to baker‘s yeast bread made without fortification, the addition of ABP slightly decreased the specific volume, especially at 10%. Nevertheless, fortification with 5% of Fermented-ABP did not interfere with the specific volume, unlike what happened with Raw-ABP. Both the addition of ABP and fermentation increased the water absorption and stability of the dough, and, at the same time, decreased its degree of softening. Many fibers present in fruit and vegetables are hydrophilic and have a natural WRC (e.g., acting as gums and hydrocolloids), which makes them ideal candidates to increase the viscosity and physical quality of dough systems. Hydrophilic fibers of fruits and vegetables, mainly pectin as in the case of ABP, minimize the structural negative effects. As a valuable biotechnology and very simple pre-treatment, fermentation of ABP with yeast and lactic acid increases the hydrophilicity of fibers, which resulted in high water absorption. Based on farninograph analysis, a dough with relevant content of DF displays an initial peak because the rapid water absorption. Further, the dough relaxes and the elastic gluten network rightly develops, which leads to a second farinograph peak. Although DF addition causes a lower kneading stability, fermentation restored the optimal dough performance. Because the crumb temperature does not exceed 100°C during bread baking, the Maillard reaction does not occur. Therefore, ingredients mainly determine the breadcrumb color. Compared to baker‘s yeast bread without addition, fermented ABP did not determine variations of the crust color.

Alcohols, aldehydes, ketones, heterocyclic compounds, and esters mainly contribute to VOCs profile of bread. Compared to the baker’s yeast bread, breads fortified with ABP contained higher levels of these compounds. These levels, in particular those of the alcohols, were proportional to ABP addition. Ethanol and isoamyl alcohol, followed by phenylethyl alcohol, were the most representative among alcohols. Isoamyl alcohol is important for the crumb flavor, giving balsamic, alcoholic, and malty aromas. It derives from the leucine catabolism by *S. cerevisiae*, according to Ehrlich pathway. Overall, the addition of ABP stimulated the yeast fermentation for all breads since the increased supply of glucose, fructose and free amino acids. Nevertheless, the addition of Fermented-ABP increased also the levels of aldehydes, esters and ketones. In particular, benzaldehyde (almond-like and nutty odors) and nonanal (citrus and floral odor) were at the highest levels. These compounds are key-odorants in sourdough breads, and are released by both lactic acid bacteria and yeasts. Sourdough yeasts release esters of long-chain unsaturated fatty acids as signaling molecules in response to lactic acid bacteria or their metabolites (Guerzoni et al., 2007). This would explain the highest level of ethyl caprylate in the bread fortified with Fermented-ABP. Ethyl caprylate possess pleasant sweet and fruity odors (Birch et al., 2013).

Dietary fibers slow down many processes associated with the digestion of starch and other glycemic carbohydrates, such as gastric emptying, small intestinal transit and transport from the lumen to mucosal surface. Therefore, DF lowers GI, which decreases the risk of developing type 2 diabetes and improves the insulin/glucose metabolism in type 2 diabetes patients (Prückler et al., 2015). Insoluble DF have the main role, representing a physical obstacle for glucose, with its consequent entrapment within the DF network. ABP are excellent sources of DF, also including a relevant content of insoluble fibers (cellulose and hemicellulose) (Gómez and Martinez, 2017). Compared to Raw-ABP and baker‘s yeast bread without addition, the bread fortification with Fermented-ABP caused a significant decrease of the values of starch hydrolysis index. The increased concentration of DF and mainly the biological acidification, which is one of the main factors decreasing the rate of starch hydrolysis, might had been responsible for this effect.

Other favorable characteristics of the bread fortified with fermented ABP dealt with the shelf life. As already known, the efficient use of fruit and vegetable by-products may delay bread staling (Curti et al., 2016). As shown using purified fibers, this effect depends on type of fibers and their content (Gómez et al., 2003; Ronda et al., 2014). Compared to the other fortified breads and baker’s yeast bread, the fortification with 5% of Fermented-ABP gave the lowest rate of moisture loss during storage. When used at 10%, Fermented-ABP also delayed mold contamination. Fruits and vegetables naturally contain inhibitory substances (Coda et al., 2011) but yeasts and mainly lactic acid bacteria have the capability to synthesize a spectrum of antifungal compounds (e.g., peptides, short-chain fatty acids, polyols) with synergistic effect.

A laboratory panel assessed the sensory attributes of the breads. Although the bread made with Raw-ABP was agreeable, breads fortified with fermented ABP reached the highest scores for several attributes. This bread had a more complex profile, which featured with high aroma intensity, and fruitiness, sourness, toasted and nutty attributes which are typical of sourdough fermentation (Katina et al., 2006). Baker’s yeast bread made without ABP fortification resulted in a simpler aroma profile.

Fermented apple by-products are suitable ingredients to fortify the wheat bread formula. Although the fortification with dietary fibers is the focus of their use, fermented apple by-products increased hydration properties and dough stability, imparted agreeable flavor and odor attributes, decreased the hydrolysis index and delayed firming and mold contamination. Re-cycling apple by-products agrees with bio-economy and environmental sustainability concepts.

## Data Availability Statement

All datasets generated for this study are included in the article/Supplementary Material.

## Author Contributions

VC and ID carried out the experiments. PF conceived the study, administrated the project, elaborated the results, and wrote the draft of the manuscript. GG developed the methodology for volatiles investigation and carried out the analysis. SP provided frozen apple by-products and contributed to the idea conceptualization. MG was the scientific advisor and critically revised the manuscript. RD conceived the study, supervised the project, elaborated the results, and wrote the draft of the manuscript. All authors read and approved the final manuscript.

## Conflict of Interest

SP was employed by the company PAN Surgelati Srl (Bolzano, Italy). The remaining authors declare that the research was conducted in the absence of any commercial or financial relationships that could be construed as a potential conflict of interest.
