# Supplementary material for: Lactic Acid Fermentation to Re-cycle Apple By-Products for Wheat Bread Fortification
Source: Front Microbiol. 2019 Nov 6;10:2574. doi: 10.3389/fmicb.2019.02574 (PMC6851242; doi:10.3389/fmicb.2019.02574)

Fig. S1. Cell density of lactic acid bacteria (black bars) and yeasts (gray bars) during incubation at 30°C of chemically acidified apples by-products (CA-ABP) and ABP inoculated with selected (single and binary cultures) *Weissella cibaria* PEP23F, *Leuconostoc mesenteroides* KI6, *Lactobacillus plantarum* 3DM, *Saccharomyces cerevisiae* AN6Y19 and *Hanseniaspora uvarum* AN8Y2C. Data are the means (±SD) of three independent experiments analyzed in triplicate.

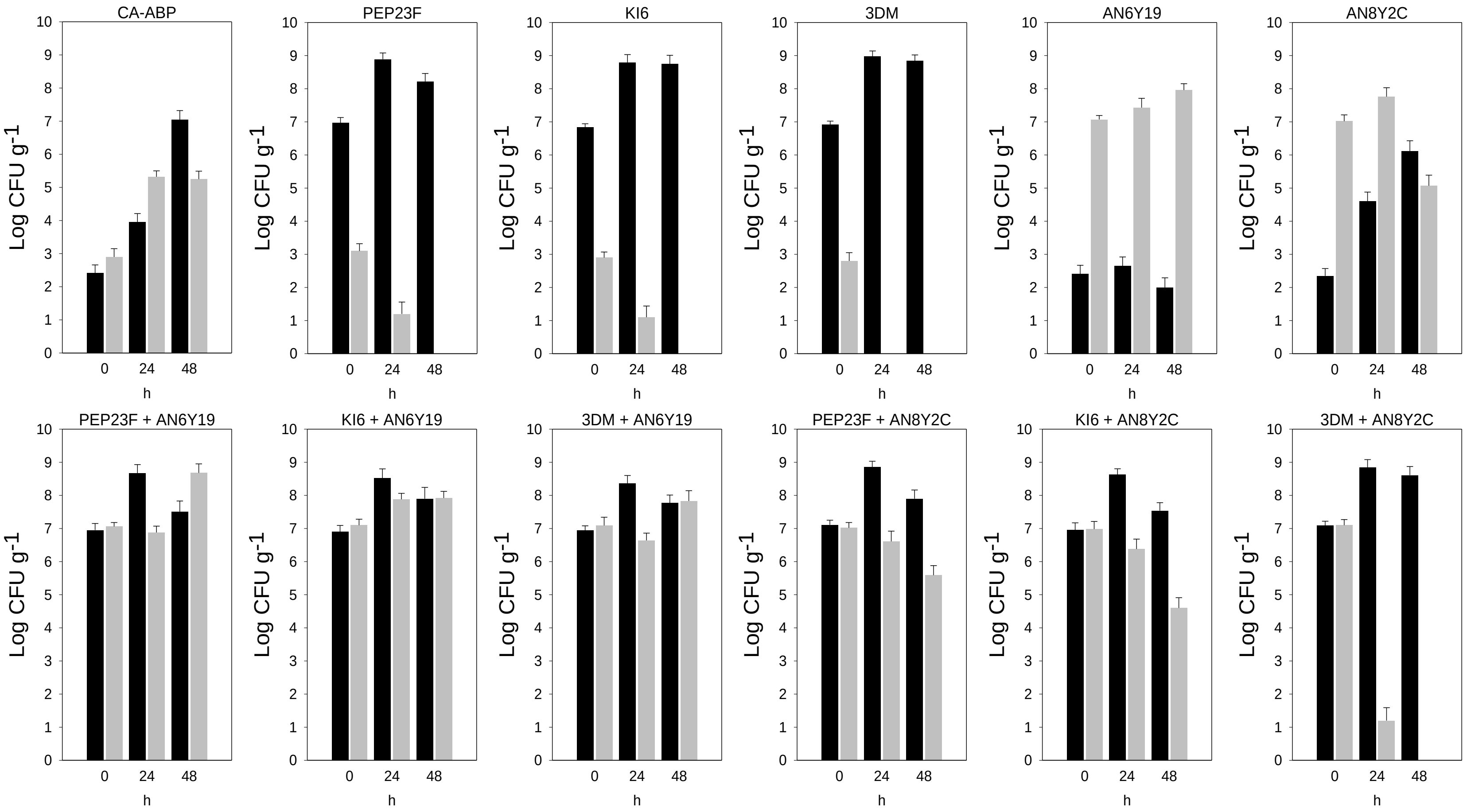

Supplement: Supplementary file 1 [file Image_1.pdf]
