# Supplementary material for: Lactic Acid Fermentation to Re-cycle Apple By-Products for Wheat Bread Fortification
Source: Front Microbiol. 2019 Nov 6;10:2574. doi: 10.3389/fmicb.2019.02574 (PMC6851242; doi:10.3389/fmicb.2019.02574)
Supplement: Supplementary file 2 [file Image_2.pdf]

Fig. S2. Representative randomly amplified polymorphic DNA-PCR (RAPD-PCR) profiles of lactic acid bacteria (obtained with M13 primer) and yeasts (obtained with M13m primer). RAPD-PCR profiles obtained from independent cultures of *Lactobacillus plantarum* 3DM (profile 1), *Leuconostoc mesenteroides* KI6 (profile 5), *Weissella cibaria* PEP23F (profile 9), *Saccharomyces cerevisiae* AN6Y19 (profile 13) and *Hanseniaspora uvarum* AN8Y2C (profile 18) were compared to profiles obtained from representatives isolates of lactic acid bacteria and yeasts isolated from ABP fermented (30°C for 48 h) by single cultures of *L. plantarum* 3DM (profile 2), *L. mesenteroides* KI6 (profile 6), *W. cibaria* PEP23F (profile 10), *S. cerevisiae* AN6Y19 (profile 14), or *H. uvarum* AN8Y2C (profile 19), or by binary cultures of *L. plantarum* 3DM plus *S. cerevisiae* AN6Y19 (profiles 3 and 15), *L. plantarum* 3DM plus *H. uvarum* AN8Y2C (profile 4), *L. mesenteroides* KI6 plus *S. cerevisiae* AN6Y19 (profiles 7 and 16), *L. mesenteroides* KI6 plus *H. uvarum* AN8Y2C (profiles 8 and 20), *W. cibaria* PEP23F plus *S. cerevisiae* AN6Y19 (profiles 11 and 17), or *W. cibaria* PEP23F plus *H. uvarum* AN8Y2C (profiles 12 and 21). A DNA molecular size standard was used (S).

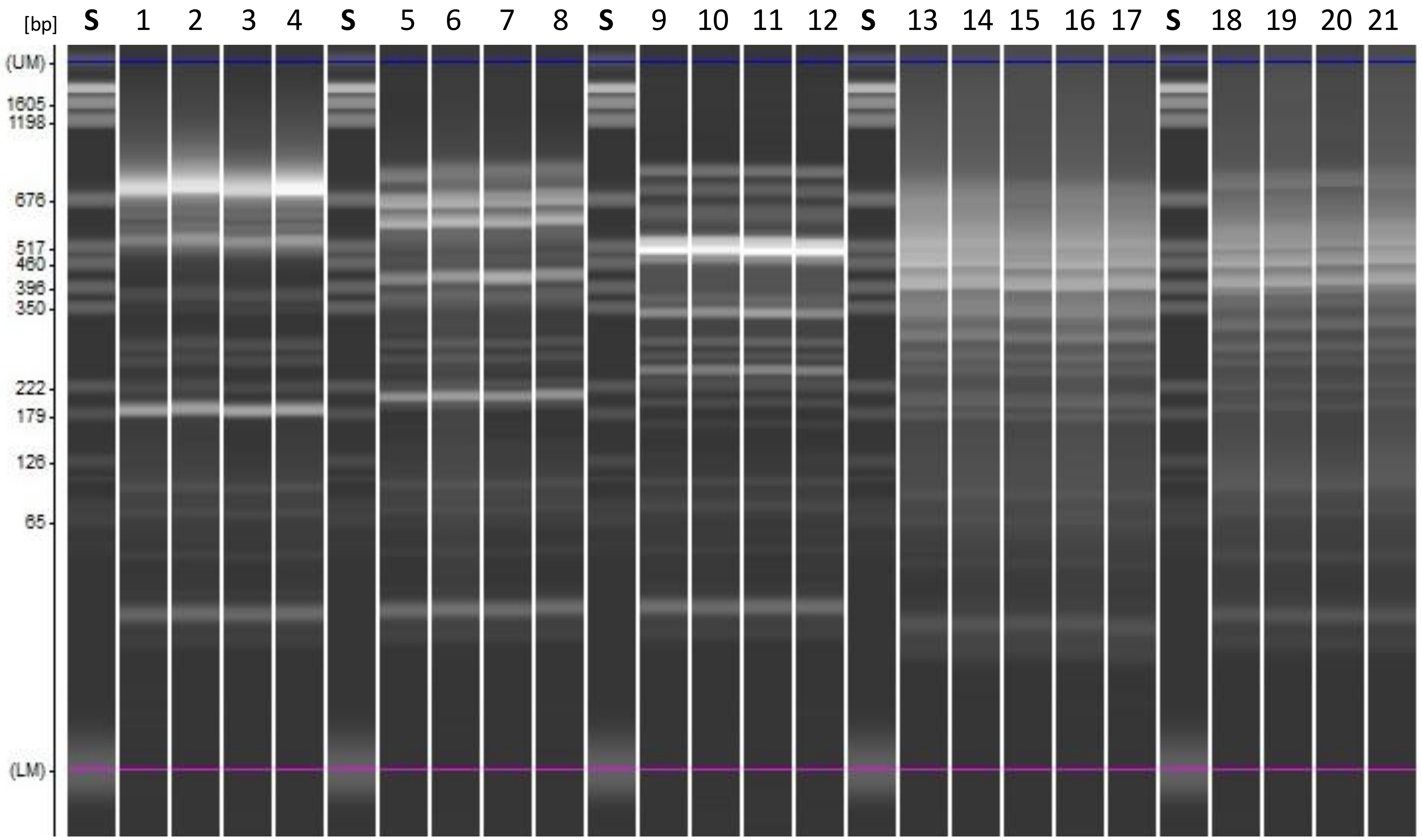

[bp]
S 1 2 3 4 S 5 6 7 8 S 9 10 11 12 S 13 14 15 16 17 S 18 19 20 21
(UM)
1605
1198
676
517
460
396
350
222
179
126
65
(LM)
