# Supplementary material for: Lactic Acid Fermentation to Re-cycle Apple By-Products for Wheat Bread Fortification
Source: Front Microbiol. 2019 Nov 6;10:2574. doi: 10.3389/fmicb.2019.02574 (PMC6851242; doi:10.3389/fmicb.2019.02574)

Fig. S3. Representative images of sections of bread manufactured with wheat flour fortified with apple by–products (ABP). Raw ABP (Raw-ABP), and chemically acidified ABP (CA-ABP) and Fermented-ABP, which were previously incubated at 30°C for 48 h. Fermentation (Fermented-ABP) was with selected binary culture of *Weissella cibaria* PEP23F and *Saccharomyces cerevisiae* AN6Y19. Raw-, CA- and Fermented-ABP were added at concentrations of 5 and 10% (w w^{-1} of flour). Baker's yeast wheat bread (BY), manufactured without addition of ABP, was the control. Digital images of bread showing the original images (A, C, G, K, E, I, and M) and computed binary images resulting from gray level thresholding at the two-cluster (B, D, H, L, F, J, and N) were provided.

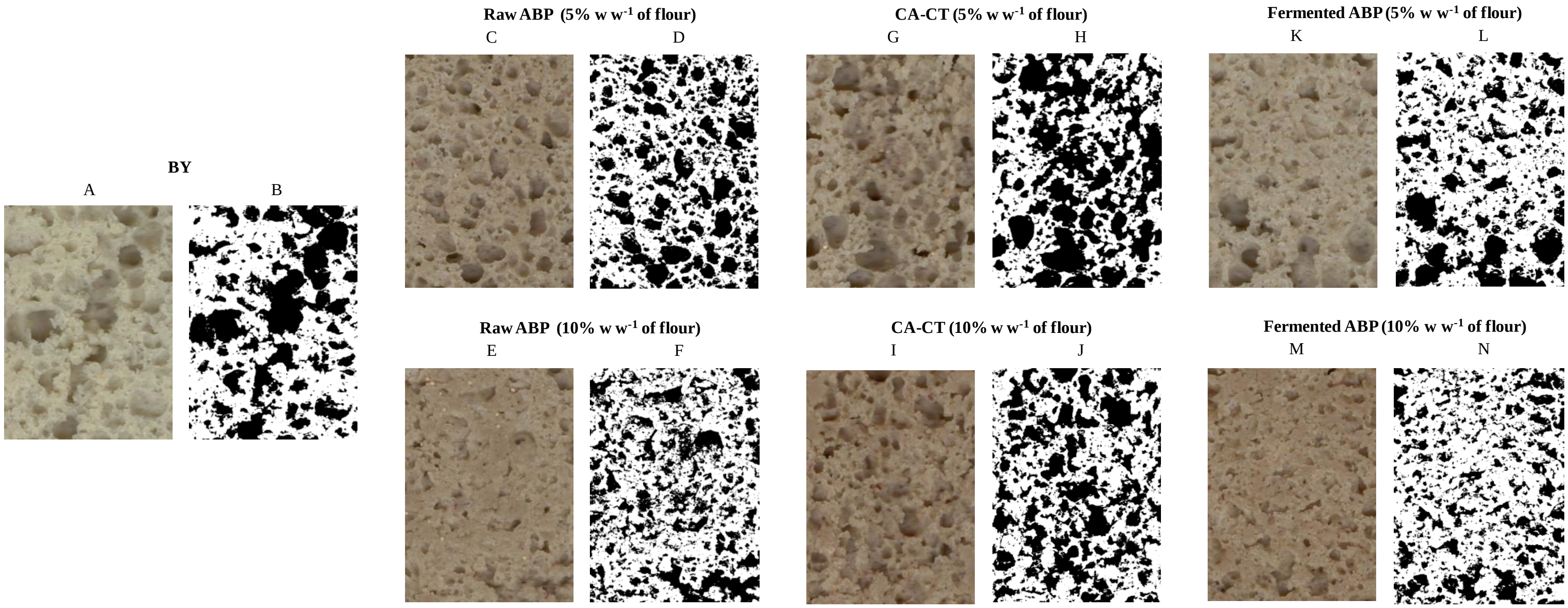

Supplement: Supplementary file 3 [file Image_3.pdf]
