# Supplementary material for: Lactic Acid Fermentation to Re-cycle Apple By-Products for Wheat Bread Fortification
Source: Front Microbiol. 2019 Nov 6;10:2574. doi: 10.3389/fmicb.2019.02574 (PMC6851242; doi:10.3389/fmicb.2019.02574)
Supplement: Supplementary file 4 [file Table_1.pdf]

Table. S1. Volume, color and crumb image analysis of bread manufactured with wheat flour fortified with apple by–products (ABP). Raw ABP (Raw-ABP), and chemically acidified ABP (CA-ABP) and Fermented-ABP, which were previously incubated at 30°C for 48 h. Fermentation (Fermented-ABP) was with the selected binary culture of *Weissella cibaria* PEP23F and *Saccharomyces cerevisiae* AN6Y19. Raw-ABP, CA-ABP and Fermented-ABP were added at concentrations of 5 and 10% (w w^{-1} of flour). Baker's yeast wheat bread (BY), manufactured without addition of ABP, was the control. Data are the means (± SD) of three independent experiments analyzed in triplicate. Data were subjected to one-way ANOVA followed by Tukey's procedure at $P < 0.05$.

	**BY**	**Raw-ABP (5% w w^{-1} of flour)**	**Raw-ABP (10% w w^{-1} of flour)**	**CA-ABP (5% w w^{-1} of flour)**	**CA-ABP (10% w w^{-1} of flour)**	**Fermented-ABP (5% w w^{-1} of flour)**	**Fermented-APB (10% w w^{-1} of flour)**
**Specific volume (cm^3 g^{-1})**	2.48 ± 0.15^{a}	2.14 ± 0.15^{b}	2.12 ± 0.15^{bc}	2.42 ± 0.12^{a}	2.00 ± 0.12^{c}	2.43 ± 0.23^{ab}	2.08 ± 0.18^{bc}
**pH**	5.91 ± 0.09^{a}	5.36 ± 0.08^{b}	4.96 ± 0.12^{c}	4.72 ± 0.13^{cd}	4.23 ± 0.15^{e}	4.85 ± 0.15^{c}	4.45 ± 0.14^{de}
**Crust color analysis**							
dE^{*}_{ab}	34.7 ± 2.3^{c}	41.6 ± 1.8^{ab}	44.8 ± 1.6^{a}	36.9 ± 1.7^{c}	37.3 ± 1.8^{c}	38.8 ± 2.2^{bc}	38.0 ± 1.8^{c}
**Crumb image analysis**							
Black pixel area (%)	41.65 ± 1.6^{b}	42.06 ± 1.0^{b}	37.28 ± 1.1^{c}	52.28 ± 1.7^{a}	44.38 ± 1.4^{b}	35.76 ± 0.8^{c}	31.22 ± 0.5^{d}

$^{a–e}$ Means within the row with different letters are significantly different ($P < 0.05$).
