# Supplementary material for: Lactic Acid Fermentation to Re-cycle Apple By-Products for Wheat Bread Fortification
Source: Front Microbiol. 2019 Nov 6;10:2574. doi: 10.3389/fmicb.2019.02574 (PMC6851242; doi:10.3389/fmicb.2019.02574)
Supplement: Supplementary file 5 [file Table_2.pdf]

Table S2. Volatile compounds (ppm) in breads manufactured with wheat flour fortified with apple by-products (ABP). Raw ABP (raw-ABP), and chemically acidified ABP (CA-ABP) and ABP-fermented, which were previously incubated at 30°C for 48 h. Fermentation (ABP-fermented) was with the selected binary culture of *Weissella cibaria* PEP23F and *Saccharomyces cerevisiae* AN6Y19. Raw-, CA- and Fermented-ABP were added at concentrations of 5 and 10% (w w^{-1} of flour). Baker's yeast wheat bread (BY), manufactured without addition of ABP, was the control. Data are the means (± SD) of three independent experiments analyzed in triplicate. Data were subjected to one-way ANOVA followed by Tukey's procedure at $P < 0.05$.

	**BY**	**Raw ABP (5% w w^{-1} of flour)**	**Raw ABP (10% w w^{-1} of flour)**	**Chemically acidified ABP (5% w w^{-1} of flour)**	**Chemically acidified ABP (10% w w^{-1} of flour)**	**Fermented ABP (5% w w^{-1} of flour)**	**Fermented APB (10% w w^{-1} of flour)**
**Alcohols**							
Isopropyl alcohol	12.4 ± 2.2[b]	n.d.	n.d.	7.2 ± 1.1[b]	n.d.	24.8 ± 5.8[a]	n.d.
Ethanol	1034.1 ± 2.8[c]	743.6 ± 150.6[c]	1930 ± 59.6[b]	839 ± 60.6[c]	2394.3 ± 67.7[a]	324.3 ± 14.5[d]	2305.2 ± 310.9[ab]
1-Propanol, 2methyl	47.6 ± 12.7[b]	24.2 ± 10.6[b]	100 ± 16.5[b]	50.1 ± 3.6[b]	138.4 ± 2.9[a]	21.5 ± 11.2[a]	140.2 ± 29.5[a]
2-Propanol,1-methoxy	8.1 ± 7.4[ab]	22.1 ± 1.8[a]	n.d.	n.d.	12.1 ± 4.7[ab]	11.3 ± 10.4[ab]	2.9 ± 4.9[b]
Isoamyl alcohol	312.9 ± 27.8[c]	398.8 ± 58.1[c]	949.7 ± 82[b]	408.9 ± 26.8[c]	1475.3 ± 39[a]	386.1 ± 26.3[c]	1452.2 ± 145.5[a]
1-Pentanol	9.9 ± 3.9[c]	11.8 ± 1.7[bc]	17.3 ± 1.6[bc]	10.1 ± 0.2[c]	26 ± 4.3[a]	9.8 ± 1.4[c]	18.8 ± 3[ab]
2-Propanol,1-butoxy	n.d.	14.8 ± 25.7	n.d.	31.7 ± 5	n.d.	n.d.	n.d.
Hexanol	9.7 ± 1.2 [d]	58.9 ± 2.5[c]	110 ± 16.3[b]	43.3 ± 3.4 [cd]	184.4 ± 17[a]	51.1 ± 0.9 [c]	114.7 ± 24.9[b]
3,5-Octadine-2-ol (3E, 5E)	n.d.	n.d.	n.d.	1.6 ± 0.1	n.d.	n.d.	n.d.
1-Octen-3-ol	6.6 ± 2.7[b]	38.6 ± 15.6[a]	37.7 ± 17.5[a]	25.3 ± 11.6[ab]	30.3 ± 2.9[ab]	16.1 ± 1.8[ab]	43.7 ± 10.3[a]
2 Ethyl hexanol	12.4 ± 2.4	13.5 ± 1.4	12.4 ± 1.4	9.1 ± 2	13.4 ± 3.4	9.3 ± 1.8	14.1 ± 1.2
1-Octanol	1.6 ± 0.4[c]	2.3 ± 2.1[bc]	4.7 ± 1.3[ab]	1.6 ± 0.3	5 ± 0.6[ab]	1.9 ± 0.1[c]	6 ± 0.4[a]
2-Octen-1-ol (Z)	1.2 ± 0.2	6.6 ± 3.5	5.7 ± 3.3	3.6 ± 2	2.9 ± 2.6	1.3 ± 0.2	5.7 ± 1.5

Table S2. Continued.

	**BY**	**Raw ABP (5% w w^{-1} of flour)**	**Raw ABP (10% w w^{-1} of flour)**	**Chemically acidified ABP (5% w w^{-1} of flour)**	**Chemically acidified ABP (10% w w^{-1} of flour)**	**Fermented ABP (5% w w^{-1} of flour)**	**Fermented APB (10% w w^{-1} of flour)**
2-Furamethanol (furfuryl alcohol)	81.6 ± 15.7[a]	48.6 ± 1.2[b]	26.9 ± 11.7[bc]	20 ± 1.1[bc]	15.7 ± 4[c]	87 ± 19.4[a]	11.7 ± 2.7[c]
1-Propanolo, 3-(methylthio)- (ion 106)	1.5 ± 0.7[e]	9.6 ± 1.6[cd]	27.8 ± 6.1[b]	9.4 ± 1.8[d]	52 ± 2.3[a]	7.5 ± 0.4[de]	17.5 ± 2.6[c]
1,3-Propanediol diacetate	6.6 ± 2	21.5 ± 12.8	19.5 ± 11.7	14.5 ± 3.4	24.9 ± 8.2	15.3 ± 1	18.1 ± 1.5
Benzyl alcohol	4.5 ± 0.6[e]	64.4 ± 6.6[cd]	101.4 ± 30.2[bc]	110 ± 29.5[bc]	217.9 ± 12.8[a]	30.6 ± 0.5[de]	126.4 ± 21[b]
Phenylethyl Alcohol	243.3 ± 42.7[c]	751.8 ± 34.7[ab]	741.3 ± 211.8[ab]	708.3 ± 114.2[ab]	977.5 ± 13.6[a]	654.9 ± 9.7[b]	792.6 ± 119.1[ab]
2-Methoxy-4-vinylphenol	0.8 ± 0.2	1.4 ± 0.5	1.8 ± 0.7	1.3 ± 0.2	n.d.	1.4 ± 0.4	1.7 ± 0.4
Total alcohols	1794.5 ± 113.8[c]	2232.5 ± 150.1[c]	4086.3 ± 233.5[b]	2295 ± 206.4[c]	5570.2 ± 39.9[a]	1654.1 ± 55.4[c]	5071.6 ± 604.9[a]
**Esters**							
Ethyl acetate	111.5 ± 5.7[a]	n.d.	34.7 ± 18.6[b]	29.9 ± 8.3[bc]	8.8 ± 5.3[c]	15.8 ± 1.5[bc]	n.d.
1-Butanol,3-methyl,acetate (isoamyl acetate)	6.6 ± 6.1[b]	n.d.	2 ± 3.4[c]	13.7 ± 1.5[b]	1.9 ± 3.2[a]	n.d.	24 ± 5.2[a]
Hexanoic acid, ethyl ester (ethyl caproate)	6.9 ± 11.9[b]	15.7 ± 14.1[b]	26.2 ± 12[ab]	7.4 ± 0.8[b]	48.4 ± 7.9[a]	23.2 ± 9.9[ab]	21.7 ± 11.2[ab]
Propanoic acid, 2-hydroxy-, ethyl ester (lactic acid, ethyl ester)	n.d.	4.1 ± 0.3[c]	16.9 ± 2.4[bc]	104.8 ± 19.6[a]	32.5 ± 1[b]	2.2 ± 2[c]	92.1 ± 8.9[a]
Butanoic acid, 2-methyl-, hexyl ester/ butyl 2-methylbutanoate	n.d.	n.d.	n.d.	23.1 ± 3.8	n.d.	n.d.	n.d.
Butanoic acid,2-methyl-,hexyl ester	0.2 ± 0.4[d]	73.9 ± 12[bc]	57 ± 5.8[bc]	34.6 ± 6.2[cd]	90 ± 2.3[b]	75 ± 5.7[bc]	284 ± 40.3[a]
Octanoic acid, ethyl ester (Ethyl caprylate)	25.3 ± 11.8[de]	55.2 ± 11.7[cd]	64.2 ± 13.2[c]	42.6 ± 4.3[cde]	107.8 ± 4.7[b]	19.6 ± 2.5[e]	141.1 ± 17.6[a]

Table S2. Continued.

	BY	Raw ABP (5% w w^{-1} of flour)	Raw ABP (10% w w^{-1} of flour)	Chemically acidified ABP (5% w w^{-1} of flour)	Chemically acidified ABP (10% w w^{-1} of flour)	Fermented ABP (5% w w^{-1} of flour)	Fermented APB (10% w w^{-1} of flour)
Propanoic acid, 2-hydroxy-, 3-methylbutyl ester (isomayl lactate)	n.d.	n.d.	n.d.	19.6 ± 2.9^{b}	n.d.	n.d.	24.8 ± 3.3^{a}
Hexanoic acid, hexyl ester (hexyl ester)	n.d.	7.4 ± 1.9^{ab}	4.7 ± 4.3^{ab}	2.7 ± 0.3^{b}	6.6 ± 2^{ab}	2.6 ± 0.8^{b}	9.1 ± 1.8^{a}
Decanoic acid, ethyl ester	2.1 ± 1.5	9 ± 5.8	11 ± 6.5	3.9 ± 0.7	11.3 ± 1.6	2.3 ± 0.4	11.5 ± 1.3
Acetic acid, phenylmethyl ester	n.d.	n.d.	n.d.	1.2 ± 0.3^{b}	1.8 ± 0.1^{b}	n.d.	22.4 ± 3.4^{a}
Ethyl 4-hydroxybutanoate	0.4 ± 0.2^{b}	0.6 ± 0.7^{b}	5.3 ± 3.6^{ab}	0.9 ± 0.4^{b}	9.8 ± 2.2^{a}	2.4 ± 4.1^{b}	2 ± 0.3^{b}
Hexanedioic acid, dimethyl ester	n.d.	n.d.	n.d.	0 ± 0	n.d.	n.d.	4.7 ± 0.3
Acetic acid, phenylethyl ester	2 ± 0.01^{b}	2.9 ± 0.2^{b}	16.1 ± 3.2^{a}	5.4 ± 1.1^{b}	15.9 ± 2^{a}	2.9 ± 0.4^{b}	5.6 ± 0.7^{b}
Total esters	155.1 ± 16.8^{c}	168.8 ± 27.2^{c}	238 ± 49.3^{bc}	289.9 ± 26.6^{b}	334.9 ± 9.7^{b}	145.9 ± 17.6^{c}	642.8 ± 73.9^{a}
**Aldehydes**							
Acetaldehyde	75.6 ± 6.3^{b}	42 ± 4.4^{c}	46 ± 3.8^{c}	81.4 ± 15.2^{b}	121.8 ± 6.6^{a}	93.5 ± 7.4^{b}	89.9 ± 5.3^{b}
Butanal, 2-methyl	27.8 ± 5.3^{abc}	12.9 ± 11.5^{c}	47.1 ± 8^{abc}	20.8 ± 2.4^{b}	38.4 ± 6.7^{abc}	51.3 ± 22.7^{a}	12.2 ± 1.7^{c}
Butanal, 3-methyl	45.2 ± 9^{b}	32.5 ± 2.8^{b}	107.8 ± 10.2^{a}	50.4 ± 4^{b}	91.3 ± 11.2^{a}	83.5 ± 19.5^{a}	24.1 ± 4.6^{b}
n-Pentanal	n.d.	14.9 ± 1.8^{b}	24.1 ± 3.4^{b}	12.7 ± 3.8^{b}	21.8 ± 2.5^{b}	10.7 ± 1.2^{b}	57.6 ± 17.1^{a}
n-Hexanal (n-caproaldehyde)	105.3 ± 5.7^{a}	86.5 ± 11.7^{ab}	57.5 ± 14.4^{bc}	50.9 ± 1.2^{bc}	49.3 ± 19.5^{bc}	82.9 ± 30.3^{ab}	42.9 ± 5.6^{c}
n-Heptanal	n.d.	n.d.	8.3 ± 3.2^{ab}	10.2 ± 2.9^{ab}	12.5 ± 2.2^{ab}	6.8 ± 1.3^{b}	14.8 ± 3^{a}
Octanal	6.6 ± 0.5^{ab}	10.5 ± 3.1^{ab}	7.4 ± 5.4^{ab}	5.2 ± 0.9^{b}	5.2 ± 0.4^{b}	7.6 ± 1.7^{ab}	13.6 ± 1.6^{a}
Cis 2-Heptenal	0.7 ± 1.2^{b}	43.2 ± 24.3^{b}	28.1 ± 4.4^{b}	29.2 ± 28^{b}	43.9 ± 17.8^{b}	15.5 ± 9.4^{b}	111.9 ± 31.8^{a}
Nonanal	70.1 ± 2.7^{cd}	106.3 ± 12.8^{ab}	63.8 ± 8.6^{cd}	18.5 ± 0.6^{e}	84 ± 5.5^{bc}	52 ± 2^{d}	115.4 ± 13^{a}
Furfural	29.3 ± 1.7^{d}	175.6 ± 23.3^{a}	72 ± 10.6^{c}	64 ± 7.1^{c}	92.2 ± 9.6^{bc}	89.8 ± 6^{bc}	113.4 ± 10.9^{b}

Table S2. Continued.

	BY	Raw ABP (5% w w^{-1} of flour)	Raw ABP (10% w w^{-1} of flour)	Chemically acidified ABP (5% w w^{-1} of flour)	Chemically acidified ABP (10% w w^{-1} of flour)	Fermented ABP (5% w w^{-1} of flour)	Fermented APB (10% w w^{-1} of flour)
n-Decanal	10.5 ± 3.1^{b}	17.3 ± 1.5^{a}	8.1 ± 1.1^{b}	10.9 ± 1.5^{b}	10.9 ± 3.5^{b}	11.4 ± 1.5^{ab}	11.2 ± 1.8^{b}
Benzaldehyde	33.8 ± 6.8^{c}	91 ± 4.4^{b}	58.7 ± 17.2^{bc}	64.3 ± 15.7^{bc}	92.6 ± 2.9^{b}	66.8 ± 11^{bc}	173.7 ± 25.1^{a}
2-Nonenal (Z)	9.5 ± 1.5^{c}	29 ± 1^{a}	16.7 ± 1.3^{b}	16.2 ± 1.5^{b}	16.9 ± 1.8^{b}	16.5 ± 2^{b}	27.4 ± 2.8^{a}
Benzeneacetaldehyde	7.1 ± 0.9^{c}	17.9 ± 1.8^{ab}	21.5 ± 5.1^{ab}	14.1 ± 0.5^{bc}	23.9 ± 2.5^{a}	15.8 ± 3.2^{b}	23.7 ± 2.4^{a}
Total aldehydes	421.5 ± 30.3^{e}	679.7 ± 31.7^{bc}	567.1 ± 62.8^{d}	448.8 ± 21.1^{e}	704.7 ± 6.8^{b}	604.3 ± 47.3^{cd}	831.7 ± 11.5^{a}
**Ketones**							
2-Propanone (Acetone)	n.d.	23.1 ± 5.2	19.2 ± 0.9	10.7 ± 18.5	11.8 ± 20.4	28.6 ± 10.4	n.d.
2-Butanone	91.7 ± 3.8^{a}	n.d.	4.5 ± 7.9^{c}	24.9 ± 9.3^{b}	n.d.	n.d.	n.d.
2-Hepatanone	6 ± 5.6^{b}	60.3 ± 14.6^{a}	3.9 ± 6.8^{b}	5.3 ± 0.8^{b}	5.5 ± 1.1^{b}	4.2 ± 0.9^{b}	5.8 ± 0.5^{b}
2-Octanone	0.5 ± 0.8^{b}	2.1 ± 3.7^{ab}	n.d.	1.7 ± 0.1^{b}	n.d.	n.d.	5.8 ± 0.4^{a}
Acetoin (2-butanone, 3-hydroxy)	25.8 ± 2.9^{bc}	77.7 ± 18.1^{a}	18.1 ± 6.3^{c}	26.8 ± 1.7^{bc}	13.1 ± 2.3^{c}	40.5 ± 3^{b}	n.d.
1-Octen-3-one	n.d.	24.3 ± 15.8^{ab}	6.7 ± 11.6^{b}	5.6 ± 4^{b}	12.6 ± 8.5^{b}	1.9 ± 3.2^{b}	57.6 ± 22.9^{a}
2-Propanone,1-hydroxy(Acetol)	2.9 ± 0.9^{b}	9.1 ± 1.9^{a}	n.d.	3.7 ± 0.2^{b}	n.d.	10.6 ± 2.3^{a}	n.d.
6-Methyl-5-hepten-2-one	60.4 ± 6.7^{de}	114.2 ± 22.4^{cd}	131.1 ± 28.5^{c}	45.3 ± 4^{e}	204.7 ± 9.1^{b}	99.7 ± 7.3^{cde}	277.2 ± 38.2^{a}
2-Hydroxy-3-pentanone	3.1 ± 0.1^{b}	4 ± 1.7^{ab}	n.d.	3.3 ± 0.2^{b}	n.d.	5.3 ± 0.2^{a}	n.d.
4-Octanone,5-hydroxy-2,7-dimethyl	n.d.	7.1 ± 1.1^{ab}	4 ± 1.8^{c}	3.1 ± 0.3^{c}	3.5 ± 0.3^{c}	4.6 ± 0.3^{bc}	8.5 ± 1.4^{a}
3,5,5-Trimethyl-2-cyclohexenone	0.3 ± 0.5^{a}	n.d.	21.9 ± 5.9^{b}	n.d.	n.d.	n.d.	46 ± 7.1^{a}
2(3H)-Furanone,dihydro-(butyrolactone)	4 ± 0.9^{b}	5 ± 0.4^{b}	7 ± 2.7^{ab}	3.7 ± 0.9^{b}	10.5 ± 1.7^{a}	5.8 ± 0.8^{b}	5.6 ± 1^{b}
Total ketones	194.6 ± 7.6^{cd}	326.8 ± 34.1^{b}	216.4 ± 35.5^{c}	134.1 ± 21.5^{d}	261.8 ± 25.9^{bc}	201 ± 17.4^{cd}	406.5 ± 27.7^{a}

Table S2. Continued.

	BY	Raw ABP (5% w w^{-1} of flour)	Raw ABP (10% w w^{-1} of flour)	Chemically acidified ABP (5% w w^{-1} of flour)	Chemically acidified ABP (10% w w^{-1} of flour)	Fermented ABP (5% w w^{-1} of flour)	Fermented APB (10% w w^{-1} of flour)
**Carboxylic acids**							
Acetic acid	n.d.	6 ± 4^{ab}	9.7 ± 3.1^{a}	2.9 ± 0.6^{b}	4.9 ± 3^{ab}	3.9 ± 1.9^{ab}	2.9 ± 0.2^{b}
2 + 3 Methylbutanoic acid (coeluizione)	9.1 ± 0.8^{c}	19.7 ± 5.9^{bc}	36.3 ± 9.7^{bc}	18.3 ± 1.6^{bc}	66.3 ± 9.3^{a}	27.6 ± 21.7^{bc}	38.9 ± 4.4^{ab}
4-Bromopyrazole-3(5)-carboxylic acid	n.d.	n.d.	n.d.	0.7 ± 0.3	n.d.	n.d.	n.d.
Hexanoic acid	1.1 ± 0.4^{b}	22.3 ± 3.8^{a}	16.3 ± 3.2^{a}	3.7 ± 0.5^{b}	15.1 ± 5.5^{a}	5.8 ± 3.6^{b}	5.2 ± 2^{b}
Heptanoic acid	0.6 ± 0.4	2.0 ± 1.0	4.4 ± 3.3	1.9 ± 0.1	3.1 ± 1.8	1.4 ± 0	2.4 ± 0.2
Octanoic acid	4.3 ± 3.9^{b}	5 ± 2^{ab}	13.7 ± 5.2^{a}	4.1 ± 0.8^{b}	9.5 ± 3.9^{ab}	3.4 ± 0.7^{b}	6.1 ± 3.4^{ab}
Nonanoic acid	10.4 ± 2.5^{c}	16 ± 4.6^{abc}	25.2 ± 4.9^{a}	12.2 ± 0.7^{bc}	22.1 ± 2.5^{a}	17.3 ± 3.6^{abc}	20.7 ± 3.7^{ab}
Decanoic acid	1.1 ± 1.1^{b}	3.3 ± 0.5^{a}	2 ± 0.9^{ab}	0.7 ± 0.2^{b}	1.6 ± 1.1^{ab}	0.8 ± 0.1^{b}	1.6 ± 0.3^{ab}
Total carboxylic acids	26.6 ± 6.4^{d}	74.3 ± 9^{abcd}	107.7 ± 24.2^{ab}	44.5 ± 3.9^{cd}	122.6 ± 26.3^{a}	60.1 ± 27.6^{bcd}	77.8 ± 13^{abc}
**Heterocyclic compounds**							
Pyrazine (p-diazine)	9.1 ± 2.9^{b}	n.d.	9.3 ± 2.6^{b}	5.8 ± 1.3^{b}	n.d.	n.d.	28.3 ± 4.5^{a}
Furan, 2-penthyl	19.5 ± 11.4	19.5 ± 6.5	35.5 ± 10.7	30.4 ± 7.7	41 ± 10.7	23.5 ± 5.8	27.9 ± 17.6
Methylpyrazine	22.7 ± 7^{ab}	11.8 ± 1.5^{c}	13.8 ± 4.2^{bc}	12.5 ± 2^{bc}	3.6 ± 0.6^{c}	28.9 ± 3.8^{a}	15.2 ± 3.3^{bc}
Pyrazine, 2,6-dimethyl-	3.1 ± 0.1^{b}	n.d.	3.1 ± 5.4^{b}	4.3 ± 1.2^{b}	n.d.	13 ± 3.9^{a}	n.d.
Pyrazine, trimethyl	2.8 ± 0.7^{b}	16.9 ± 9.9^{b}	12.2 ± 6.4^{b}	3.3 ± 0.9^{b}	18.6 ± 2.7^{b}	6.9 ± 1.7^{b}	46.1 ± 15.5^{a}
2-Acetylfuran (ethanone, 1-(2-furanyl)-)	5.2 ± 0.6^{b}	6.2 ± 1^{ab}	3.3 ± 0.8^{c}	2.3 ± 0^{c}	n.d.	7.6 ± 0.8^{a}	2.8 ± 0.2^{c}
Pyrrole	8.6 ± 0.6^{a}	1.9 ± 1.9^{b}	4.8 ± 1^{ab}	2.6 ± 0.2^{b}	2.1 ± 1.9^{b}	4.8 ± 0.4^{ab}	1.4 ± 2.4^{b}
2(3 H)-Furanone. 5-ethyldihydro- (ion85)	2.2 ± 0.4^{ab}	2 ± 0.01^{ab}	2.3 ± 0.3^{ab}	2.2 ± 0.3^{ab}	2 ± 0.5^{ab}	2.6 ± 0.1^{a}	1.8 ± 0.3^{b}
4(H)-Pirydine, n-acetyl	0.9 ± 0.4^{c}	4.2 ± 0.7^{b}	2.4 ± 1.3^{bc}	1.6 ± 0.1^{bc}	2.2 ± 0.7^{bc}	7.3 ± 2.2^{a}	2.7 ± 0.3^{bc}
Maltol	7.4 ± 2.1	2.4 ± 1.6	4.2 ± 3.1	2 ± 1	1.8 ± 1.7	9.2 ± 6.4	1.1 ± 0.2

Table S2. Continued.

	BY	Raw ABP (5% w w^{-1} of flour)	Raw ABP (10% w w^{-1} of flour)	Chemically acidified ABP (5% w w^{-1} of flour)	Chemically acidified ABP (10% w w^{-1} of flour)	Fermented ABP (5% w w^{-1} of flour)	Fermented APB (10% w w^{-1} of flour)
Total heterocyclic compounds	81.4 ± 23.3[a]	64.9 ± 16.7[b]	90.9 ± 25.4[ab]	67 ± 10.7[b]	71.3 ± 14.5[ab]	103.8 ± 21.3[ab]	127.3 ± 26.4[a]
**Terpenes**							
d-Limonene	7.2 ± 6.3[c]	33.7 ± 5[b]	9.3 ± 1.5[c]	7.3 ± 1.1[c]	6.3 ± 2[c]	4 ± 3.7[c]	71.3 ± 6.9[a]
(Z,E)-Alpha-farnesene	0.3 ± 0.2[d]	165.3 ± 9.9[b]	159.3 ± 25.7[b]	133.7 ± 14.6[bc]	280.8 ± 6.4[a]	95.4 ± 6.8[c]	303.7 ± 38.2[a]
Total terpenes	7.5 ± 6.4[e]	198.9 ± 13[c]	168.6 ± 26.3[c]	141 ± 15.5[cd]	287.1 ± 7.6[b]	99.4 ± 10.2[d]	375 ± 45.1[a]
**Alkanes**							
Hexane	87.6 ± 10.5[a]	43.4 ± 9.3[e]	46.1 ± 5.2[de]	63.4 ± 4.3[bcde]	78.9 ± 5.4[ab]	71.2 ± 4.6[abc]	54.5 ± 6.7[cd]
Octane	5 ± 8.7[e]	83.8 ± 12.2[b]	107 ± 4[abc]	64.1 ± 10.5[cd]	140.7 ± 29[a]	41.7 ± 14.9[de]	88.7 ± 10.4[bc]
Total alkanes	92.6 ± 19.2[c]	127.2 ± 15.4[bc]	153 ± 9[b]	127.5 ± 14.4[bc]	219.5 ± 34.4[a]	112.8 ± 17.2[bc]	143.2 ± 15.9[bc]
**Total volatiles**	2774 ± 203[d]	3873 ± 207[c]	5628 ± 446[b]	3548 ± 257[cd]	7572 ± 94[a]	2982 ± 130[cd]	7676 ± 778[a]

n.d., not detected.

[a–e] Means within the row with different letters are significantly different ($P < 0.05$).
