# Supplementary material for: Lactic Acid Fermentation to Re-cycle Apple By-Products for Wheat Bread Fortification
Source: Front Microbiol. 2019 Nov 6;10:2574. doi: 10.3389/fmicb.2019.02574 (PMC6851242; doi:10.3389/fmicb.2019.02574)
Supplement: Supplementary file 6 [file Table_3.pdf]

Table S3. Mold contamination during 14 days of incubation at room temperature of bread slices manufactured with wheat flour fortified with apple by-products (ABP). Raw ABP (raw-ABP), and chemically acidified ABP (CA-ABP) and ABP-fermented, which were previously incubated at 30°C for 48 h. Fermentation (ABP-fermented) was with the selected binary culture of *Weissella cibaria* PEP23F and *Saccharomyces cerevisiae* AN6Y19. Raw-, CA- and Fermented-ABP were added at concentrations of 5 and 10% (w w^{-1} of flour). Baker's yeast wheat bread (BY), manufactured without addition of ABP, was the control. Data are the means (± SD) of three independent experiments analyzed in triplicate. Data were subjected to one-way ANOVA followed by Tukey's procedure at $P < 0.05$.

	**Moulds contamination (%)***						
**Storage (days)**	**BY**	**Raw-ABP (5% w w^{-1} of flour)**	**Raw-ABP (10% w w^{-1} of flour)**	**CA-ABP (5% w w^{-1} of flour)**	**CA-ABP (10% w w^{-1} of flour)**	**Fermented-ABP (5% w w^{-1} of flour)**	**Fermented-APB (10% w w^{-1} of flour)**
11	–	–	–	–	–	–	–
12	+	+	+	+	+	+	+
13	+	+	++	+	+	+	+
14	++++	++++	+++++	++++	+++	+++	+

*Contamination was determined as the percentage of the surface covered by the visible micelia of molds, scored as follows: –, 0% contamination of the slice surface; +, 1–5% contamination of the slice surface; ++, 6–10 % contamination of the slice surface; +++, 11–20 % contamination of the slice surface; ++++, 21–30% contamination of the slice surface; +++++, 31 – 40% contamination of the slice surface.
